# From Host Response to Microbiological Risk: Association of the RIN-IM Score with Culture-Positive Sepsis and Antimicrobial Resistance in Critically Ill Adults

**DOI:** 10.3390/diagnostics16203350

**Published:** 2026-10-14

**Authors:** Davut Ipek, Yusuf Ipek, Recep Dokuyucu

**Affiliations:** 1 Department of Infectious Diseases and Clinical Microbiology, School of Medicine, Mardin Artuklu University, 47000 Mardin, Türkiye; 2 Department of Anesthesiology and Reanimation, Mardin Training and Research Hospital, 47100 Mardin, Türkiye; dr.y.ipek40@gmail.com; 3 Department of Physiology, Medical Specialization Training Center (TUSMER), 06230 Ankara, Türkiye; drecepfatih@gmail.com

**Keywords:** RIN-IM score, sepsis, culture positivity, antimicrobial resistance, multidrug resistance, intensive care unit, inflammatory indices, risk stratification

## Abstract

**Background/Objectives:** Early identification of microbiologically confirmed sepsis and antimicrobial-resistant infection remains challenging in critically ill patients. The renal–inflammatory–nutritional–metabolic (RIN-IM) score integrates serum creatinine, the systemic immune-inflammation index, pan-immune inflammation value, serum albumin, and triglyceride–glucose index. This study investigated the association of the RIN-IM score with culture-positive sepsis and antimicrobial resistance phenotypes in critically ill adults. **Methods:** This single-center retrospective cohort study included 318 adults with sepsis admitted to the intensive care unit between 1 January 2023 and 31 March 2026. The RIN-IM score was calculated from laboratory measurements obtained within 24 h and categorized as low (0–3), intermediate (4–6), or high risk (7–10). The primary outcome was culture-positive sepsis. Secondary analyses, restricted to patients with culture-positive sepsis, examined associations between RIN-IM and multidrug-resistant (MDR), extensively drug-resistant (XDR), and pandrug-resistant phenotypes. **Results:** Culture-positive sepsis was identified in 154 patients (48.4%). The median RIN-IM score was higher in culture-positive than in culture-negative patients (6 [interquartile range, 4–8] vs. 4 [2–5]; *p* < 0.001). Culture positivity increased from 29.1% in the low-risk group to 50.4% in the intermediate-risk group and 74.6% in the high-risk group. Each one-point increase in RIN-IM was independently associated with culture-positive sepsis (adjusted odds ratio [aOR], 1.35; 95% confidence interval [CI], 1.21–1.51), while high- versus low-risk classification was associated with an aOR of 4.65 (95% CI, 2.25–9.62). Among culture-positive patients, 67 (43.5%) had MDR, and 20 (13.0%) had XDR infections. Each one-point score increase was independently associated with MDR infection (aOR, 1.29; 95% CI, 1.12–1.48). The exploratory XDR analysis was based on 20 events and used Firth’s bias-reduced logistic regression; a one-point score increase was associated with XDR infection (aOR, 1.29; 95% CI, 1.09–1.52), but the categorical estimates were imprecise. The score demonstrated moderate discrimination for culture-positive sepsis (AUC, 0.752; 95% CI, 0.699–0.805) and MDR infection (AUC, 0.724; 95% CI, 0.643–0.805); discrimination for XDR infection was exploratory (AUC, 0.742; 95% CI, 0.635–0.849). Twenty-eight-day mortality increased from 13.6% to 27.7% and 47.9% across the three risk categories (*p* < 0.001). **Conclusions:** Higher admission RIN-IM scores were associated with culture-positive sepsis. Among patients with culture-positive sepsis, higher scores were also associated with MDR infection; this subgroup analysis was conditioned on microbiological confirmation and should not be interpreted as prediction of resistant infection in the overall sepsis population. The association with XDR infection was exploratory, based on only 20 events, and requires confirmation in larger independent cohorts. Because RIN-IM assessment and microbiological sampling were not fully time-synchronized, these findings should not be interpreted as real-time microbiological prediction or evidence of clinical utility. RIN-IM should not replace culture-based diagnosis, susceptibility testing, individual risk-factor assessment, local antibiograms, or clinical judgment. The observed associations do not establish a direct biological relationship between RIN-IM and microbial burden or antimicrobial resistance.

## 1. Introduction

Sepsis is a life-threatening syndrome characterized by acute organ dysfunction resulting from a dysregulated host response to infection [1]. Despite advances in critical care, antimicrobial therapy, and organ-support strategies, sepsis remains a major global health burden. The most recent Global Burden of Disease analysis showed that the worldwide burden of sepsis increased during 2020–2021, reversing the reductions observed during the preceding decades, with particularly marked increases among older adults [2]. The heterogeneity of the host response, infection source, causative pathogen, antimicrobial susceptibility, and underlying comorbidities continues to complicate early diagnosis and individualized treatment.

Microbiological confirmation is central to the management of sepsis because identification of the causative pathogen and its antimicrobial susceptibility profile enables targeted treatment, supports antimicrobial de-escalation, and assists with source-control decisions. However, pathogen recovery remains incomplete, even among patients who fulfil the Sepsis-3 criteria. Culture yield is influenced by infection site, pathogen burden, sampling quality, fastidious organisms, and prior antimicrobial exposure. In a prospective cohort study, blood-culture positivity was substantially lower when cultures were obtained during antimicrobial treatment than when they were collected before treatment [3]. At the same time, antimicrobial treatment should not be delayed in patients with a high likelihood of sepsis or septic shock. The 2026 Surviving Sepsis Campaign guidelines therefore emphasize timely treatment together with responsible antimicrobial use, risk-adapted empirical coverage for multidrug-resistant pathogens, and de-escalation when microbiological and susceptibility results become available [4].

Culture-positive and culture-negative sepsis may also represent clinically distinct phenotypes. A systematic review and meta-analysis found no consistent difference in mortality between these groups, although culture-positive sepsis was associated with longer hospital stay and duration of mechanical ventilation [5]. More recently, a prospective nationwide multicentre cohort demonstrated that culture-positive sepsis was associated with greater initial illness severity and a different pattern of organ dysfunction, whereas culture-negative sepsis was more frequently associated with pulmonary infection and respiratory failure [6]. These findings suggest that culture positivity is not merely a diagnostic result but may identify a subgroup with distinct pathogen, host-response, and clinical characteristics. Nevertheless, reliable admission-based tools for estimating the probability of microbiological confirmation remain limited.

Antimicrobial resistance further complicates this clinical uncertainty. In 2021, bacterial antimicrobial resistance was associated with an estimated 4.71 million deaths worldwide, highlighting its growing effect on global morbidity and mortality [7]. This problem is particularly important in intensive care units, where critical illness, invasive devices, prolonged hospitalization, repeated antibiotic exposure, and cross-transmission increase the likelihood of infection with resistant organisms. In culture-proven community-onset sepsis, inadequate empirical therapy directed against resistant pathogens has been associated with worse outcomes; conversely, unnecessary broad-spectrum treatment may expose patients to avoidable toxicity and promote further resistance [8]. Individual epidemiological and clinical risk factors, including previous antimicrobial exposure, healthcare contact, colonization history, infection source, and local resistance patterns, remain central to assessment of resistance risk. Whether an early host-response profile is associated with subsequent microbiological confirmation or resistance phenotypes has not been established.

Risk stratification in sepsis has traditionally relied on physiological severity scores and individual biomarkers. However, single variables may inadequately represent the interconnected renal, immune-inflammatory, nutritional, and metabolic disturbances occurring during critical illness. The renal–inflammatory–nutritional–metabolic (RIN-IM) score was developed as a pragmatic composite of admission serum creatinine, the systemic immune-inflammation index (SII), the pan-immune inflammation value (PIV), serum albumin, and the triglyceride–glucose (TyG) index [9]. These routinely available measurements may collectively reflect the severity of systemic inflammation, organ dysfunction, metabolic stress, and impaired nutritional reserve. SII, PIV, TyG, creatinine, and albumin have each been associated with adverse outcomes in sepsis and critical illness [10,11,12,13].

Any observed association between a higher RIN-IM score and microbiological confirmation may reflect shared relationships with systemic inflammation, organ dysfunction, infection source, healthcare exposure, prior antimicrobial treatment, and microbiological sampling practices rather than a direct relationship with microbial burden. Likewise, an association with MDR or XDR phenotypes may reflect the clustering of severe illness with healthcare exposure, invasive devices, prolonged hospitalization, prior antimicrobial treatment, and infection source rather than detection of pathogens or resistance mechanisms. RIN-IM does not include microbiological, epidemiological, or treatment-exposure variables and cannot identify viable organisms, quantify microbial burden, determine antimicrobial-resistance mechanisms, or establish patient-specific susceptibility patterns. Thus, its relationship with culture positivity and antimicrobial resistance should be regarded as an observational association with host-response severity and microbiological confirmation, not as evidence of direct biological or microbiological prediction.

To our knowledge, no study has evaluated whether the admission RIN-IM score is associated with episode-level culture positivity or antimicrobial-resistance phenotypes in critically ill adults with sepsis. Accordingly, the primary aim of this study was to examine the association between admission RIN-IM score and culture-positive sepsis. Secondary aims were to describe its associations with MDR, XDR, and PDR phenotypes among culture-positive patients and to characterize causative pathogens and specimen types. We hypothesized that higher RIN-IM scores would be associated with a greater probability of microbiological confirmation and, within the culture-positive subgroup, with resistance phenotypes. These analyses were exploratory and were not designed to establish microbiological prediction or clinical utility.

## 2. Materials and Methods

### 2.1. Study Design and Setting

This retrospective, observational cohort study was conducted in the Anesthesiology and Reanimation Intensive Care Unit of Mardin Training and Research Hospital, Türkiye. Consecutive adult patients admitted between 1 January 2023 and 31 March 2026 were screened for eligibility. The study was designed and reported in accordance with the Strengthening the Reporting of Observational Studies in Epidemiology (STROBE) recommendations.

The study protocol was approved by the Non-Interventional Clinical Research Ethics Committee of Mardin Artuklu University (Decision No. 2026/7-42, Meeting No. 7; 2 July 2026). The requirement for written informed consent was waived by the ethics committee because of the retrospective study design and the exclusive use of routinely collected clinical data. All data were anonymized before analysis, and the study was conducted in accordance with the principles of the Declaration of Helsinki.

The present cohort was distinct from the cohort used for the development of the RIN-IM score by Kayan et al. The development and internal-validation study was conducted in Ankara at a different hospital, whereas the present study used consecutive sepsis-specific ICU records from Mardin Training and Research Hospital. Therefore, no patient, hospitalization episode, or patient-level record overlapped between the two datasets. Although R.D. is a co-author of the original RIN-IM development study, the shared authorship did not involve reuse of patient-level data or duplicate reporting.

### 2.2. Study Population

Patients were eligible if they were aged ≥18 years, had suspected or confirmed infection during their ICU stay, fulfilled the Sepsis-3 criteria, and had at least one microbiological specimen obtained during the index sepsis episode. Sepsis was defined as suspected or documented infection accompanied by an acute increase of ≥2 points in the Sequential Organ Failure Assessment (SOFA) score [1]. This pragmatic approach was applied because reliable pre-infection organ-function data were not uniformly available in the retrospective records; its potential to misclassify chronic dysfunction as acute organ dysfunction is acknowledged as a study limitation.

The index sepsis episode was defined as the first episode meeting the eligibility criteria during the ICU admission. The index time was the first documented time at which the patient fulfilled the Sepsis-3 criteria. RIN-IM was calculated from the earliest eligible laboratory measurements obtained within the first 24 h after ICU admission and was not recalculated at the index sepsis time. Therefore, the analysis evaluated the association between an early ICU-admission host-response profile and episode-level microbiological confirmation, rather than a time-synchronized prediction of culture positivity. Microbiological specimens obtained from 48 h before to 72 h after the index time were considered related to the index episode.

Patients were excluded if they were younger than 18 years, did not fulfil the Sepsis-3 criteria, had no interpretable microbiological results, or lacked one or more laboratory measurements required to calculate the RIN-IM Score. For patients with multiple ICU admissions or recurrent sepsis episodes during the same hospitalization, only the first eligible episode was included. Specimens considered to represent contamination or colonization without clinical evidence of infection were not classified as culture-positive sepsis. Because all consecutive patients meeting the eligibility criteria were included, no formal a priori sample-size calculation was performed. Among the 449 admissions meeting the Sepsis-3 criteria, 131 were excluded because no valid microbiological specimen related to the index episode was available (n = 49), one or more RIN-IM components were missing (n = 38), the admission represented a repeat ICU admission (n = 27), or essential clinical or microbiological records were incomplete (n = 17).

### 2.3. Data Collection

Data were retrospectively obtained from the hospital information management system, electronic ICU records, laboratory information system, medication-administration records, and microbiology laboratory database and were entered into a standardized data-collection form after removal of direct patient identifiers. The recorded variables included age; sex; ICU admission diagnosis and source; infection source; community- or hospital-onset infection; invasive-device use; and major comorbidities, including diabetes mellitus, chronic kidney disease, chronic cardiovascular or respiratory disease, malignancy, and immunosuppression. Illness severity was assessed using the SOFA score and, when available, the Acute Physiology and Chronic Health Evaluation II score. Vasopressor use, invasive mechanical ventilation, and serum lactate concentrations were also recorded. Laboratory variables comprised complete blood count parameters, C-reactive protein, procalcitonin when available, serum creatinine, albumin, glucose, triglycerides, and other routinely measured biochemical parameters. For calculation of the RIN-IM Score, the earliest eligible laboratory measurements obtained within the first 24 h after ICU admission were used. The RIN-IM score was therefore treated as an ICU-admission measurement and was not recalculated at the index sepsis time. Accordingly, the primary analysis evaluated the association between an early ICU-admission host-response profile and episode-level culture positivity, rather than a time-synchronized prediction of microbiological positivity. Antimicrobial-treatment data included antimicrobial exposure before culture collection, the empirical antimicrobial regimen, and the timing of culture collection relative to antimicrobial initiation. Microbiological data included specimen type, collection time, isolated microorganism, and antimicrobial susceptibility results. The recorded clinical outcomes were 28-day all-cause mortality, ICU mortality, in-hospital mortality, duration of invasive mechanical ventilation, and ICU and hospital lengths of stay. Antimicrobial exposure before culture collection was defined as receipt of at least one dose of a systemic antibacterial agent within the preceding 48 h, whereas hospital-onset infection was defined as an index infection occurring more than 48 h after hospital admission.

### 2.4. Calculation of the RIN-IM Score

The RIN-IM Score was calculated according to its original development algorithm [9]. The score integrates five routinely available laboratory variables representing four complementary physiological domains: serum creatinine for renal dysfunction; the systemic immune-inflammation index (SII) and pan-immune inflammation value (PIV) for systemic inflammatory and immune-cell imbalance; serum albumin for nutritional, protein, and inflammatory status; and the triglyceride–glucose (TyG) index for insulin-related metabolic stress. The five components had been prespecified before development of the original model based on their biological relevance, reported prognostic associations in critically ill populations, and availability during early ICU assessment [10,11,12,13]. In the present study, the original scoring algorithm was applied without re-estimating coefficients, modifying component thresholds, or redefining the risk categories.

During the original development process, all five predictors were initially examined as continuous variables. Because SII and PIV typically exhibit markedly right-skewed distributions, these variables were evaluated after natural-log transformation. Potential nonlinear relationships with mortality were investigated using restricted cubic spline functions with three knots placed at the 10th, 50th, and 90th percentiles. Categorization was undertaken only after examination of the continuous risk functions, because premature categorization of continuous predictors may result in information loss, reduced statistical power, and unstable sample-dependent thresholds [14]. The thresholds were therefore not selected through separate single-predictor receiver operating characteristic analyses or by maximizing the Youden index. The stability of the development procedure, including predictor transformation, assessment of functional form, threshold identification, and score simplification, was evaluated using 1000 bootstrap resamples [15].

For the present analysis, the earliest eligible laboratory measurements obtained after ICU admission and within the first 24 h were used. Serum creatinine and albumin were obtained from the earliest available biochemical panel. SII and PIV were calculated from the same complete blood count to ensure temporal consistency among neutrophil, lymphocyte, monocyte, and platelet measurements. The TyG index was calculated using the earliest available triglyceride and glucose concentrations recorded within the first 24 h after ICU admission: TyG index = ln[(triglycerides [mg/dL] × glucose [mg/dL])/2]. Because samples were obtained during emergency and critical-care assessment, fasting status was neither required nor assumed and could not be consistently verified from the retrospective records. Likewise, same-phlebotomy sampling and timing relative to early metabolic interventions could not be uniformly confirmed.

Each component was assigned 0, 1, or 2 points according to the predefined thresholds. Serum creatinine values of <1.50, 1.50–1.99, and ≥2.00 mg/dL were assigned 0, 1, and 2 points, respectively. The corresponding SII categories were <1000, 1000–2999, and ≥3000, while the PIV categories were <500, 500–1199, and ≥1200. Serum albumin concentrations of ≥3.50, 2.50–3.49, and <2.50 g/dL were assigned 0, 1, and 2 points, respectively. TyG index values of <9.00, 9.00–9.49, and ≥9.50 were assigned 0, 1, and 2 points. The complete calculation algorithm is presented in Table 1.

The resulting total score ranged from 0 to 10. Total scores were classified as low (0–3 points), intermediate (4–6 points), or high (7–10 points). These categories represent a pragmatic bedside simplification of the original continuous model. The equal 0-, 1-, and 2-point assignments were based on ordered risk gradients and broadly comparable standardized associations across the five components; they were not obtained by directly rounding regression coefficients. The three total-score categories were not selected using outcome-specific ROC analysis or prespecified clinical-action thresholds and should therefore be interpreted as descriptive risk strata.

The serum creatinine thresholds used in the score were not derived from kidney disease: Improving Global Outcomes acute kidney injury stages and were not intended to diagnose or classify acute kidney injury. Likewise, the SII, PIV, and TyG thresholds should not be regarded as universal clinical cut-offs because published values vary across ICU populations [16,17,18]. SOFA and APACHE II scores were evaluated as reference measures of illness severity but were not components of the RIN-IM Score. C-reactive protein, procalcitonin, admission glucose, triglycerides, and other inflammatory or metabolic variables were not assigned separate points; glucose and triglyceride concentrations contributed to the score exclusively through calculation of the TyG index. Patients lacking any of the five required components within the predefined 24-h window were considered to have a non-calculable RIN-IM Score and were excluded from the primary score-based analyses.

### 2.5. Definition of Culture-Positive Sepsis

A positive microbiological result was classified as clinically significant only when it was considered compatible with the documented index sepsis episode. Adjudication was performed independently by two investigators (D.I., an infectious diseases and clinical microbiology physician, and Y.I., an intensive care physician) using a prespecified review framework that incorporated the specimen source, contemporaneous clinical and radiological findings when available, the treating team’s documented infection assessment, and the plausibility of the isolated organism as a cause of infection at that site. Organisms considered contaminants or colonizers in the absence of supporting clinical evidence were classified as culture-negative. Discordant assessments were resolved by consensus after joint review of the complete source record. Adjudication was completed before linkage with the analytical dataset; the reviewers did not have access to the calculated RIN-IM total score or risk category. However, because routinely collected laboratory results formed part of the clinical record reviewed for adjudication, the reviewers were not blinded to individual laboratory values that could contribute to RIN-IM. Formal inter-rater agreement was not calculated.

### 2.6. Antimicrobial Resistance Definitions

Antimicrobial susceptibility testing was performed in the hospital microbiology laboratory using routine laboratory methods. Minimum inhibitory concentration and/or disk-diffusion results were interpreted according to the European Committee on Antimicrobial Susceptibility Testing (EUCAST) clinical breakpoint tables applicable at the time of testing (versions 13.0–16.0, as applicable during the study period). Isolates were categorized as susceptible (S); susceptible, increased exposure (I); or resistant (R). The final laboratory-reported susceptibility category was used for the present retrospective analysis; results were not retrospectively reinterpreted using a single later-version breakpoint table. Antimicrobial resistance phenotypes were classified at the isolate level according to the internationally standardized definitions proposed by Magiorakos et al. [19]. Multidrug resistance (MDR) was defined as acquired non-susceptibility to at least one antimicrobial agent in three or more antimicrobial categories. Extensively drug-resistant (XDR) was defined as acquired non-susceptibility to at least one antimicrobial agent in all but two or fewer relevant antimicrobial categories; thus, XDR isolates remained susceptible to agents in no more than two categories. Pandrug resistance (PDR) was defined as non-susceptibility to all tested agents in all antimicrobial categories considered appropriate for the respective microorganism. Only organism-specific antimicrobial categories were included, and intrinsic resistance was not considered acquired resistance. Isolates for which an insufficient number of antimicrobial categories had been tested were not classified as XDR or PDR unless the available susceptibility profile allowed a definitive assignment. When multiple isolates of the same species with an identical resistance phenotype were recovered from the same patient during the same septic episode, only the first isolate was included in the resistance analysis to prevent duplicate counting.

### 2.7. Study Outcomes

The primary outcome was culture-positive sepsis, defined as the recovery of at least one clinically significant bacterial or fungal pathogen from a microbiological specimen obtained within the predefined sepsis-related sampling window. The primary analysis evaluated the association between the RIN-IM score, assessed both as a continuous variable and according to the predefined low-, intermediate-, and high-risk categories, and culture positivity. Secondary outcomes included the prevalence and distribution of multidrug-resistant (MDR), extensively drug-resistant (XDR), and pandrug-resistant (PDR) organisms among culture-positive cases; the association of RIN-IM with MDR and XDR phenotypes was evaluated only within this culture-positive subgroup and was considered exploratory. Additional clinical outcomes were 28-day, ICU, and in-hospital mortality; requirement for invasive mechanical ventilation and vasopressor support; duration of mechanical ventilation; and lengths of ICU and hospital stay. The discriminatory performance of the RIN-IM score for identifying culture-positive sepsis and resistant-organism infections was evaluated using receiver operating characteristic curve analysis.

### 2.8. Statistical Analysis

All statistical analyses were performed using IBM SPSS Statistics version 27.0 (IBM Corp., Armonk, NY, USA) and R software (version 4.3.0; R Foundation for Statistical Computing, Vienna, Austria). The distribution of continuous variables was evaluated using histograms, Q–Q plots, and the Shapiro–Wilk test. Normally distributed variables were expressed as mean ± standard deviation and compared using the independent-samples t-test or one-way analysis of variance, whereas non-normally distributed variables were presented as median and interquartile range and compared using the Mann–Whitney U test or Kruskal–Wallis test, as appropriate. Categorical variables were reported as numbers and percentages. Pearson’s chi-square test was used only when expected cell frequencies were adequate; otherwise, Fisher’s exact test was used for 2 × 2 tables and the Fisher–Freeman–Halton exact test was used for larger sparse contingency tables. Isolate-level microbiological and resistance data were summarized descriptively only. Comparisons were performed between culture-positive and culture-negative patients and across the predefined low-, intermediate-, and high-risk RIN-IM categories. Trends in culture positivity and antimicrobial resistance across increasing RIN-IM categories were assessed using the Cochran–Armitage trend test. The primary association between the RIN-IM score and culture-positive sepsis was evaluated using binary logistic regression. The score was examined separately as a continuous variable, expressed per one-point increase, and as a categorical variable, with the low-risk group as the reference category. Multivariable models were adjusted for prespecified clinically relevant covariates, including age, sex, antimicrobial exposure before culture collection, hospital-onset infection, immunosuppression, infection source, and illness severity. Because serum creatinine is a component of the RIN-IM score and also contributes to the renal domain of the SOFA score, the non-renal SOFA score was used in the primary adjusted model to reduce mathematical coupling; total SOFA was evaluated in a sensitivity analysis. To quantify redundancy between the inflammatory components, the association between SII and PIV was assessed using Spearman’s correlation coefficient. In an exploratory component-level logistic regression model including ln(SII) and ln(PIV), variance inflation factors (VIFs) and tolerance statistics were calculated. These diagnostics were intended to characterize collinearity between the constituent inflammatory indices and were not used to replace the prespecified primary models, in which only the total RIN-IM score, rather than its individual components, was entered. To reduce confounding, covariates were prespecified on clinical grounds and included age, sex, pre-culture antimicrobial exposure, hospital-onset infection, immunosuppression, infection source, and non-renal SOFA score. Sensitivity analyses examined alternative severity adjustment using total SOFA and APACHE II, restriction to patients without pre-culture antimicrobial exposure, stratification by infection onset, and exclusion of fungal infections. Residual confounding from unmeasured or incompletely recorded healthcare exposures, prior antimicrobial treatment, sampling practices, and colonization history could not be eliminated in this retrospective design. Covariates were selected on clinical grounds rather than according to univariable *p*-values, and the number of variables included was restricted according to the number of outcome events. Isolate-level organism distributions, antimicrobial susceptibility results, and resistance phenotypes were summarized descriptively only. No hypothesis tests or regression analyses were performed at the isolate level because some patients contributed more than one isolate and independence of observations could not be assumed. Inferential analyses of MDR and XDR were conducted only at the patient level, with each culture-positive patient counted once. Firth’s bias-reduced logistic regression was used for sparse outcomes or when complete or quasi-complete separation occurred. Results were reported as odds ratios with 95% confidence intervals. Discrimination of the RIN-IM score for culture-positive sepsis and resistant-organism infection was assessed using receiver operating characteristic curves and the area under the curve with 95% confidence intervals. To assess whether RIN-IM provided incremental prognostic information beyond routinely available clinical variables and illness severity, we compared a prespecified clinical reference model with an extended model additionally including the continuous RIN-IM score. The reference model included age, sex, antimicrobial exposure before culture collection, hospital-onset infection, immunosuppression, infection source, and non-renal SOFA score. Non-renal SOFA was used in the primary comparison because creatinine contributes to both the renal SOFA domain and the RIN-IM score. Incremental performance was evaluated using the likelihood-ratio test, apparent and bootstrap-corrected area under the receiver operating characteristic curve (AUC), the DeLong test for correlated AUCs, Brier score, calibration intercept, and calibration slope. As a sensitivity analysis, the same comparison was repeated with total SOFA replacing non-renal SOFA. These analyses assessed within-cohort incremental model performance and were not intended to establish clinical utility or support score-guided management. An exploratory optimal cut-off value was determined using the Youden index, and the corresponding sensitivity, specificity, positive predictive value, and negative predictive value were calculated. The RIN-IM score, its component definitions, and its point thresholds were fixed according to the previously published development study and were not recalculated, recalibrated, or re-optimized in the present cohort. Accordingly, discrimination of the fixed RIN-IM score alone was reported as an apparent AUC with a 95% confidence interval. Bootstrap-based optimism correction was applied only to the newly fitted clinical reference and clinical-plus-RIN-IM models used for the incremental-performance analysis. In each of 1000 bootstrap samples, patients were sampled with replacement, the model intercept and all regression coefficients were refitted, and model performance was evaluated both in the bootstrap sample and in the original cohort. Mean optimism was calculated as the difference between bootstrap-sample and original-cohort performance and was subtracted from the apparent performance estimate. The RIN-IM score, its component thresholds, and the predefined risk-category cutoffs were not refitted, recalculated, or selected in any bootstrap sample. Patients lacking any component required for RIN-IM calculation were excluded from score-based analyses, and the extent of missing data was reported for each variable. No formal a priori sample-size calculation was performed because of the retrospective design; all patients meeting the eligibility criteria during the study period were included. All tests were two-sided, and a *p*-value < 0.05 was considered statistically significant; analyses of secondary outcomes were considered exploratory. Discrimination and calibration analyses were used to describe model performance within the present cohort and were not intended to establish prospective predictive utility, clinical utility, or benefit from score-guided management.

## 3. Results

**Figure 1:**
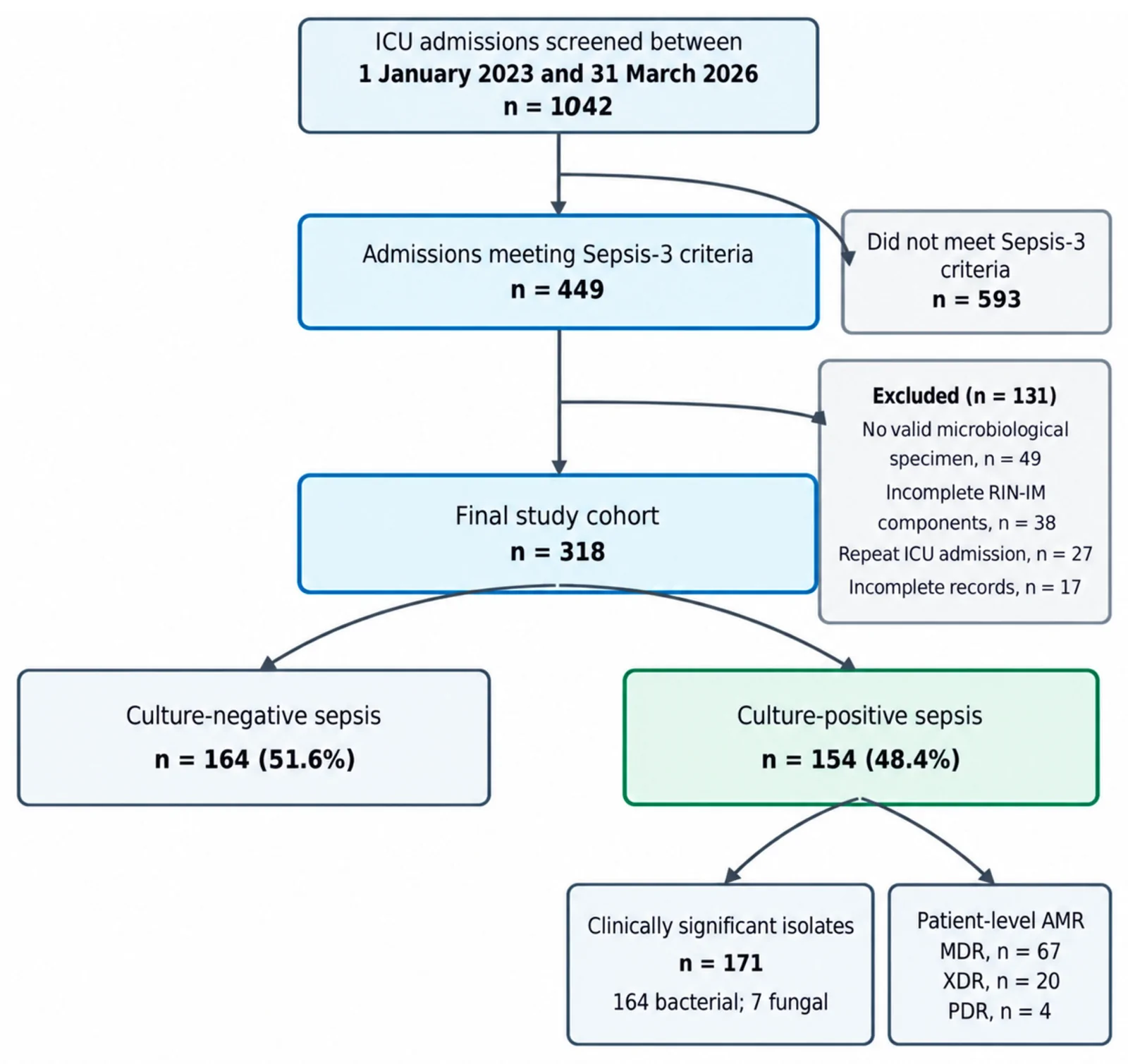
Flow diagram of patient selection and inclusion in the study.

Among 1042 ICU admissions screened between 1 January 2023 and 31 March 2026, 449 admissions met the Sepsis-3 criteria. Of these, 131 patients were excluded because no valid microbiological specimen related to the index sepsis episode was available (n = 49), one or more RIN-IM score components were incomplete (n = 38), the admission represented a repeat ICU admission (n = 27), or essential clinical or microbiological records were incomplete (n = 17). The final study cohort comprised 318 patients, including 164 (51.6%) with culture-negative sepsis and 154 (48.4%) with culture-positive sepsis (Figure 1).

Data completeness was high, and all variables required for calculating the RIN-IM score were available for the final study population. APACHE II scores were unavailable for 27 patients (8.5%); therefore, analyses involving APACHE II were performed using available cases (Supplementary Table S1).

Among the 318 patients, 164 (51.6%) were culture-negative and 154 (48.4%) were culture-positive. Age and sex distribution did not differ significantly between the groups. Compared with culture-negative patients, culture-positive patients more frequently had hospital-onset infection (48.7% vs. 35.4%, *p* = 0.016), a central venous catheter (68.2% vs. 56.1%, *p* = 0.027), immunosuppression (18.8% vs. 11.0%, *p* = 0.049), and septic shock (43.5% vs. 31.7%, *p* = 0.030), whereas antimicrobial exposure before culture collection was less frequent (45.5% vs. 65.2%, *p* < 0.001). The distribution of suspected infection sources differed between the groups (*p* = 0.046); respiratory sources were less frequent (33.1% vs. 47.6%), while urinary (22.1% vs. 18.3%), intra-abdominal (17.5% vs. 11.6%), and bloodstream- or catheter-related sources (11.7% vs. 4.9%) were more frequent among culture-positive patients. Culture-positive patients had higher SOFA scores (9 [6–12] vs. 7 [5–10], *p* < 0.001), nonrenal SOFA scores (7 [5–10] vs. 6 [4–8], *p* = 0.002), APACHE II scores (21 [15–27] vs. 18 [13–23], *p* = 0.004), and serum lactate levels (3.1 [1.8–5.2] vs. 2.4 [1.5–4.1] mmol/L, *p* = 0.015). The prevalence of invasive mechanical ventilation and the remaining comorbid conditions did not differ significantly between the groups (Table 2).

**Figure 2:**
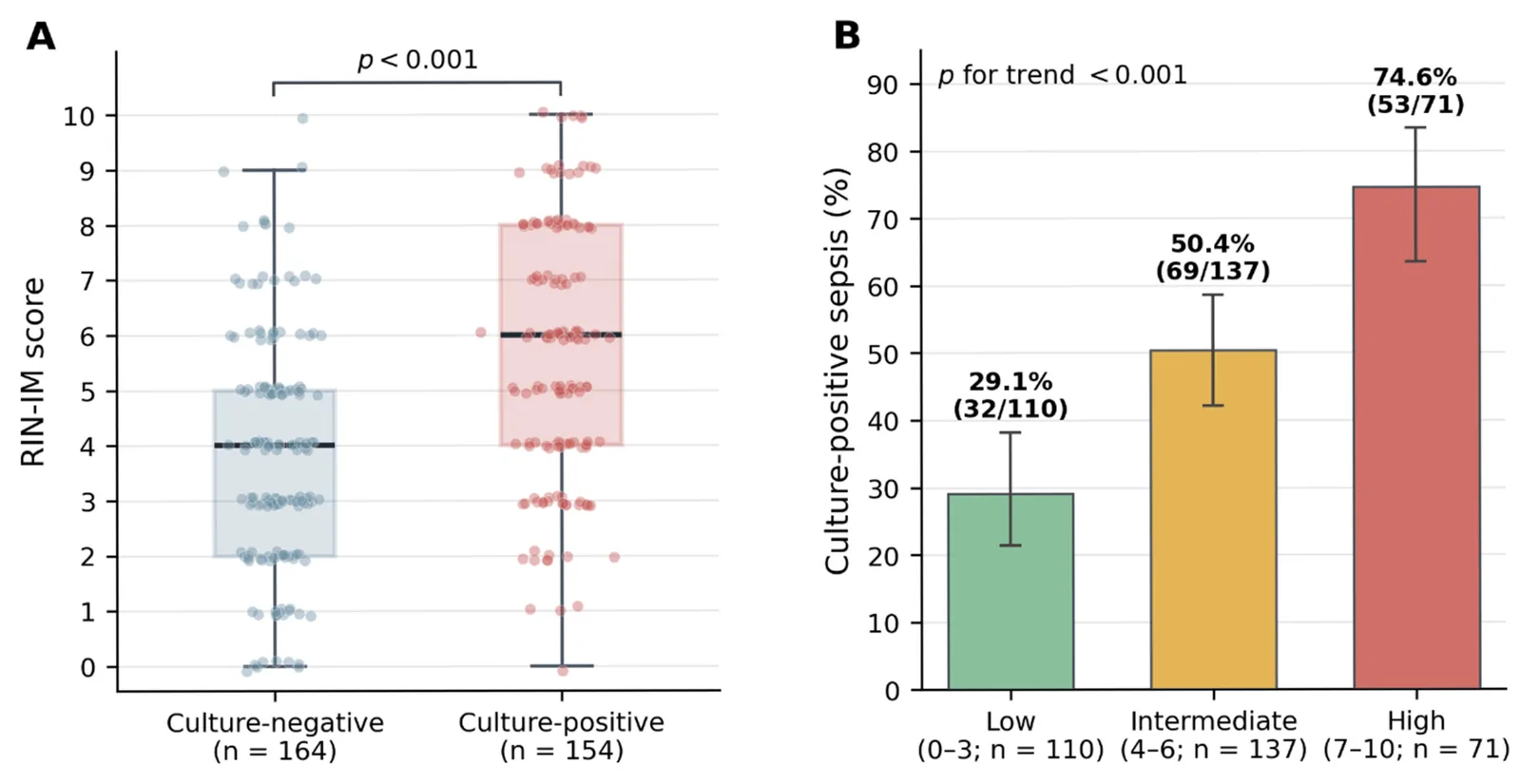
Distribution of RIN-IM scores and prevalence of culture-positive sepsis across the predefined risk categories. (**A**) Box-and-jitter plot showing RIN-IM scores in patients with culture-negative and culture-positive sepsis. Boxes represent the median and interquartile range, whiskers extend to 1.5 times the interquartile range, and individual points represent patients. (**B**) Prevalence of culture-positive sepsis in the low- (0–3), intermediate- (4–6), and high-risk (7–10) RIN-IM categories. Error bars represent 95% Wilson confidence intervals. The prevalence increased from 29.1% in the low-risk category to 50.4% in the intermediate-risk category and 74.6% in the high-risk category (*p* for trend < 0.001).

Culture-positive patients had higher white blood cell counts (15.1 [10.3–20.8] vs. 12.6 [8.8–17.2] ×10^9^/L, *p* = 0.001), neutrophil counts (13.0 [8.7–18.4] vs. 10.4 [6.9–14.7] ×10^9^/L, *p* < 0.001), and monocyte counts (0.71 [0.46–1.01] vs. 0.61 [0.39–0.87] ×10^9^/L, *p* = 0.018) but lower lymphocyte counts (0.80 [0.52–1.20] vs. 1.08 [0.73–1.51] ×10^9^/L, *p* < 0.001) than culture-negative patients. Platelet counts did not differ significantly (*p* = 0.276). Culture-positive patients also had higher creatinine (1.64 [1.09–2.46] vs. 1.26 [0.90–1.82] mg/dL, *p* = 0.002), glucose (171 [128–239] vs. 145 [113–198] mg/dL, *p* = 0.006), and triglyceride concentrations (165 [117–226] vs. 139 [101–189] mg/dL, *p* = 0.004), whereas albumin was lower (2.72 [2.30–3.17] vs. 3.12 [2.72–3.55] g/dL, *p* < 0.001). SII (3825 [2210–6910] vs. 2110 [1090–3910]), PIV (2680 [1370–5290] vs. 1280 [590–2540]), and the TyG index (9.55 [9.15–9.91] vs. 9.19 [8.84–9.57]) were higher in the culture-positive group (all *p* < 0.001). All five RIN-IM component scores were higher among culture-positive patients, and the total RIN-IM score was 6 [4–8], compared with 4 [2–5] in culture-negative patients (*p* < 0.001). The proportions of culture-positive patients classified as low, intermediate, and high risk were 20.8%, 44.8%, and 34.4%, respectively, compared with 47.6%, 41.5%, and 11.0% among culture-negative patients (*p* < 0.001). Culture-positive sepsis was present in 29.1%, 50.4%, and 74.6% of patients in the low-, intermediate-, and high-risk categories, respectively (*p* for trend < 0.001) (Table 3, Figure 2).

SII and PIV were strongly positively correlated (Spearman’s ρ = 0.840, *p* < 0.001). In the exploratory component-level logistic regression model, ln(SII) and ln(PIV) showed VIF values of 3.72 and 3.68, with tolerance values of 0.269 and 0.272, respectively (Supplementary Table S5). Thus, the inflammatory indices showed measurable overlap but did not exceed the prespecified thresholds for potentially important multicollinearity. In the primary analyses, the total RIN-IM score was entered as a composite variable; SII and PIV were not simultaneously modeled as separate covariates.

**Figure 3:**
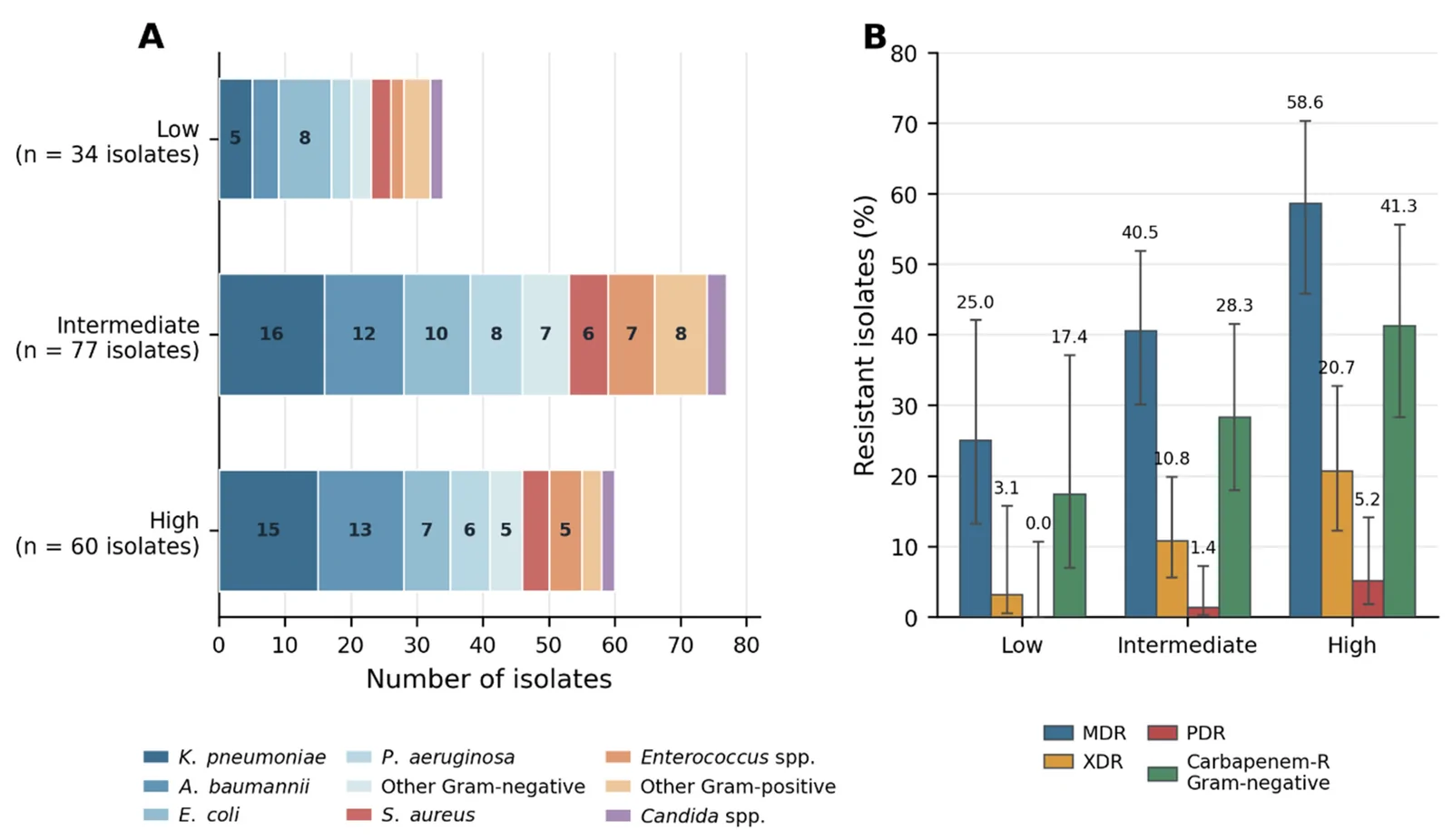
Isolate-level distribution of bacterial isolates and antimicrobial-resistance phenotypes. (**A**) Distribution of bacterial species across the low-, intermediate-, and high-risk groups; numbers within the stacked bars indicate isolate counts for the corresponding bacterial species. (**B**) Prevalence of multidrug-resistant (MDR), extensively drug-resistant (XDR), pandrug-resistant (PDR), and carbapenem-resistant Gram-negative isolates across the low-, intermediate-, and high-risk groups; error bars represent 95% confidence intervals. Percentages for MDR, XDR, and PDR refer to 164 bacterial isolates, whereas carbapenem resistance is presented among Gram-negative isolates only. Resistance phenotypes are not mutually exclusive.

Primary positive specimen type was compared across RIN-IM risk categories using the Fisher–Freeman–Halton exact test (*p* = 0.858), and polymicrobial infection frequency was evaluated using an exact test (*p* = 0.686). Among the 164 bacterial isolates, MDR, including XDR and PDR isolates, was recorded in 8 of 32 (25.0%) low-risk, 30 of 74 (40.5%) intermediate-risk, and 34 of 58 (58.6%) high-risk isolates. The corresponding numbers of XDR, including PDR, and isolates were 1 (3.1%), 8 (10.8%), and 12 (20.7%), respectively. PDR was identified in four isolates overall. Carbapenem resistance was identified in 4 of 23 (17.4%), 15 of 53 (28.3%), and 19 of 46 (41.3%) Gram-negative isolates across the low-, intermediate-, and high-risk categories, respectively. These isolate-level resistance data are descriptive because some patients contributed multiple isolates (Table 4, Figure 3).

Detailed organism-specific antimicrobial susceptibility results, including the numbers of isolates tested and their susceptible, increased-exposure, and resistant classifications, are provided in Supplementary Table S2.

MDR, XDR, and PDR were defined hierarchically; therefore, XDR isolates were included within the MDR category, and PDR isolates were included within both the XDR and MDR categories. Data are presented as n (%) or n/N (%). Patient-level percentages were calculated using the corresponding number of culture-positive patients. Isolate-level percentages were calculated using the total number of isolates within each RIN-IM category. Seventeen patients had polymicrobial infections, resulting in 171 nonduplicate clinically significant isolates from 154 patients.

Complete univariable logistic regression analyses for culture-positive sepsis and MDR infection, including demographic, clinical, severity-related, and treatment-related covariates, are presented in Supplementary Table S3.

The clinical reference model using non-renal SOFA had an apparent AUC of 0.748 (95% CI, 0.695–0.801), a bootstrap-corrected AUC of 0.742, a Brier score of 0.201, a calibration intercept of −0.04, and a calibration slope of 0.92. After addition of the continuous RIN-IM score, the apparent AUC was 0.799 (95% CI, 0.751–0.847), the bootstrap-corrected AUC was 0.791, the Brier score was 0.181, the calibration intercept was −0.02, and the calibration slope was 0.94; the AUC difference was 0.051 (*p* = 0.006), and the likelihood-ratio test *p*-value was <0.001. In the sensitivity analysis using total SOFA, the apparent and bootstrap-corrected AUCs were 0.753 (95% CI, 0.701–0.805) and 0.747, respectively, for the clinical reference model and 0.794 (95% CI, 0.746–0.842) and 0.787 after addition of RIN-IM. The corresponding Brier scores were 0.198 and 0.184, calibration intercepts were −0.05 and −0.03, calibration slopes were 0.91 and 0.93, and the AUC difference was 0.041 (*p* = 0.018); the likelihood-ratio test *p*-value was 0.002 (Table 5).

The association between the RIN-IM score and culture-positive sepsis remained directionally consistent across all prespecified sensitivity analyses, including adjustment for total SOFA score, adjustment for APACHE II in the available-case subset, restriction according to infection onset and prior antimicrobial exposure, and exclusion of fungal infections. Specifically, after exclusion of the seven patients with fungal culture positivity, the association between the RIN-IM score and culture-positive sepsis remained similar (aOR per one-point increase, 1.34; 95% CI, 1.20–1.50; *p* < 0.001; Supplementary Table S4).

The continuous and categorical representations of the RIN-IM score were evaluated in separate regression models and were not entered simultaneously. Odds ratios for categorical analyses were calculated using the low-risk group as the reference category.

**Figure 4:**
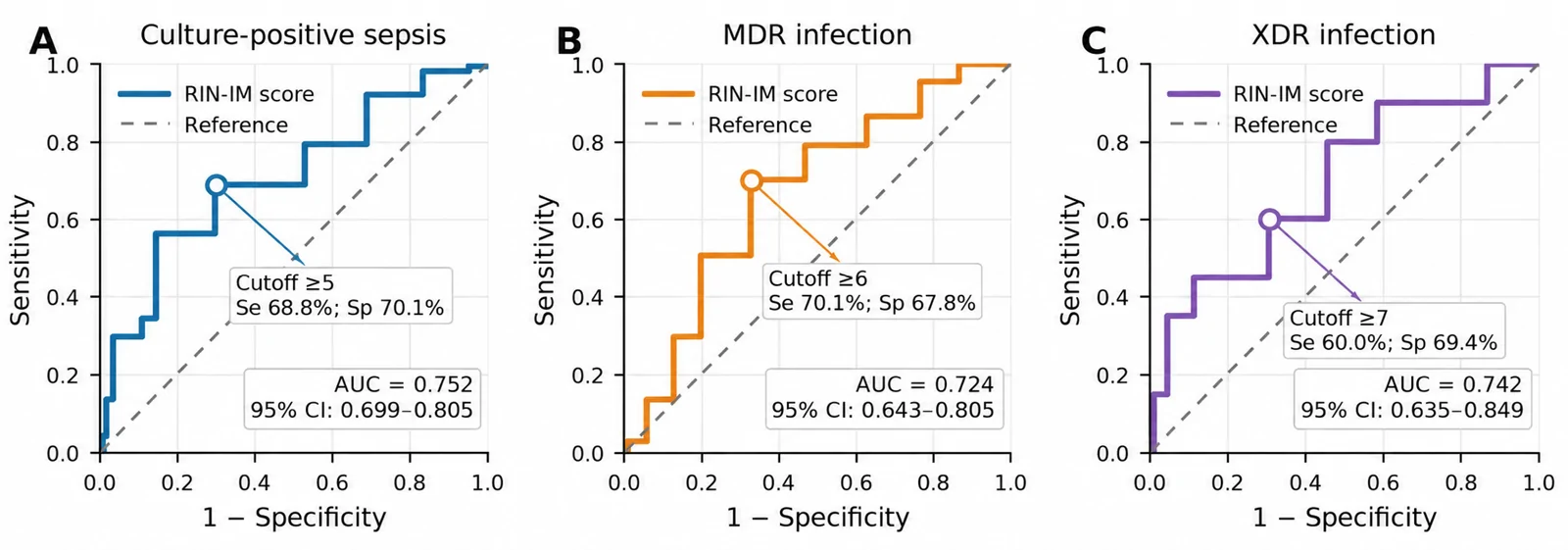
ROC curves of the RIN-IM score for culture-positive sepsis and antimicrobial-resistant infections. (**A**) Discrimination of culture-positive sepsis among 318 patients, including 154 events. (**B**) Discrimination of MDR infection among 154 culture-positive patients, including 67 events. (**C**) Exploratory discrimination of XDR infection among 154 culture-positive patients, including 20 events. Circles indicate the predefined optimal cutoff points. The AUCs were 0.752 (95% CI: 0.699–0.805), 0.724 (95% CI: 0.643–0.805), and 0.742 (95% CI: 0.635–0.849) for culture-positive sepsis, MDR infection, and XDR infection, respectively. Although the continuous RIN-IM score was associated with XDR infection, neither categorical comparison reached statistical significance: the adjusted odds ratio was 2.31 (95% CI, 0.37–14.40; *p* = 0.372) for the intermediate-risk group and 5.41 (95% CI, 0.88–33.10; *p* = 0.068) for the high-risk group versus the low-risk group. These wide confidence intervals indicate limited precision, and the XDR findings should be interpreted as exploratory.

The RIN-IM score demonstrated an apparent AUC of 0.752 (95% CI, 0.699–0.805; *p* < 0.001) for culture-positive sepsis, with a bootstrap-corrected AUC of 0.748. At the exploratory cut-off of ≥5 points, sensitivity was 68.8% (95% CI, 61.1–75.6), specificity was 70.1% (95% CI, 62.7–76.6), positive predictive value was 68.4%, and negative predictive value was 70.6%. For MDR infection, the apparent and bootstrap-corrected AUCs were 0.724 (95% CI, 0.643–0.805; *p* < 0.001) and 0.716, respectively. A cut-off of ≥6 points provided 70.1% sensitivity, 67.8% specificity, a positive predictive value of 62.7%, and a negative predictive value of 74.7%. For XDR infection, the apparent AUC was 0.742 (95% CI, 0.635–0.849; *p* < 0.001), and the bootstrap-corrected AUC was 0.729. At the ≥7-point cut-off, sensitivity was 60.0%, specificity was 69.4%, positive predictive value was 22.6%, and negative predictive value was 92.1% (Table 6, Figure 4).

Calibration analysis demonstrated generally good agreement between the probabilities predicted by the multivariable model and the observed prevalence of culture-positive sepsis (Supplementary Figure S1). The grouped observations remained close to the line of perfect calibration across the predicted-risk spectrum. After bootstrap correction, the calibration intercept was −0.04 and the calibration slope was 0.92, suggesting limited overall miscalibration and mild overfitting. The model had a Brier score of 0.194.

Bootstrap internal validation demonstrated limited optimism in the performance of the primary multivariable model. The apparent AUC decreased from 0.752 to an optimism-corrected value of 0.748. The bootstrap-corrected calibration intercept and slope were −0.04 and 0.92, respectively, while the corrected Brier score was 0.194 (Supplementary Figure S2).

Clinical outcomes differed significantly across the predefined RIN-IM risk categories. Twenty-eight-day mortality increased from 13.6% in the low-risk group to 27.7% in the intermediate-risk group and 47.9% in the high-risk group (*p* < 0.001). ICU mortality increased from 10.9% to 22.6% and 40.8%, while in-hospital mortality increased from 12.7% to 25.5% and 45.1%, respectively (both *p* < 0.001). The proportions of patients requiring invasive mechanical ventilation were 36.4%, 56.9%, and 74.6%, and the corresponding proportions requiring vasopressor support were 29.1%, 53.3%, and 77.5% across the low-, intermediate-, and high-risk groups, respectively (both *p* < 0.001). Among mechanically ventilated patients, the median duration of invasive ventilation increased from 4 [2–7] days to 7 [4–12] days and 10 [6–17] days (*p* < 0.001). Median ICU length of stay increased from 6 [4–9] to 9 [6–14] and 13 [8–21] days, while median hospital length of stay increased from 12 [8–18] to 17 [11–25] and 22 [14–33] days, respectively (both *p* < 0.001) (Table 7).

## 4. Discussion

In this study, the RIN-IM score was independently associated with culture-positive sepsis and antimicrobial-resistant infection in critically ill adults with sepsis. The prevalence of culture-positive sepsis increased progressively from 29.1% in the low-risk group to 50.4% in the intermediate-risk group and 74.6% in the high-risk group. After adjustment for demographic characteristics, pre-culture antimicrobial exposure, hospital-onset infection, immunosuppression, infection source, and nonrenal SOFA score, each one-point increase in the RIN-IM score was associated with a 35% increase in the odds of culture positivity. Patients in the high-risk group had more than fourfold higher adjusted odds of culture-positive sepsis than those in the low-risk group. A similar graded association was observed for MDR infection. Although an association with XDR infection was also observed, this analysis was based on only 20 events, required Firth’s bias-reduced logistic regression, and yielded imprecise categorical estimates; it should therefore be interpreted as exploratory. The score demonstrated moderate discriminatory ability for culture-positive sepsis, MDR infection, and XDR infection, while bootstrap correction indicated limited optimism in the primary model. Furthermore, increasing RIN-IM risk categories were accompanied by progressively higher requirements for mechanical ventilation and vasopressor support, longer ICU and hospital stays, and higher mortality. Collectively, these findings suggest that a higher RIN-IM score is associated with a more severe host-response phenotype and a greater probability of microbiological confirmation. They do not establish a distinct biological phenotype of culture-positive sepsis or indicate that the score directly reflects viable microbial burden. 

Kayan et al. originally developed and internally validated the RIN-IM model in a heterogeneous cohort of 1000 critically ill adults, using the same five laboratory domains and the same simplified 0–10-point classification employed in the present study [9]. In that development cohort, 28-day mortality increased markedly from 7.6% in the low-risk group to 27.2% in the intermediate-risk group and 77.3% in the high-risk group. Each one-point increase in the simplified RIN-IM Score was independently associated with a 68% increase in the odds of 28-day mortality. The simplified score achieved an apparent AUC of 0.890 and an optimism-corrected AUC of 0.878 and showed higher apparent discrimination than APACHE II and SOFA within the development cohort [9]. The present study extends these findings from general ICU prognostication to a sepsis-specific microbiological setting. Using the same predefined categories, culture-positive sepsis increased from 29.1% to 50.4% and 74.6%, while 28-day mortality increased from 13.6% to 27.7% and 47.9% across the low-, intermediate-, and high-risk groups, respectively. The lower mortality observed in the present high-risk group compared with the original development cohort may reflect differences in case mix, admission diagnoses, baseline severity, treatment practices, and study setting. More importantly, the present findings suggest that the biological domains captured by RIN-IM may be associated not only with short-term mortality but also with microbiological confirmation and antimicrobial-resistance phenotypes. Nevertheless, this extension should not be interpreted as evidence that RIN-IM directly identifies pathogens or resistance mechanisms. Importantly, the present cohort was assembled at a different hospital from the original RIN-IM development cohort and contained no overlapping patients or hospitalization episodes. Thus, this study represents an independent application of the score in a geographically distinct, sepsis-specific ICU population; however, it was not designed as a formal external validation of the original 28-day mortality model.

The culture-positive proportion in our cohort was 48.4%, which is consistent with the wide range reported in contemporary sepsis populations. Differences in culture positivity across studies are expected because microbiological yield depends on infection source, timing and adequacy of sampling, previous antimicrobial exposure, blood volume, local laboratory procedures, pathogen characteristics, and the inclusion or exclusion of nonblood specimens. Yang et al. reported culture positivity in 46.3% of 592 patients with sepsis, a proportion remarkably similar to that observed in our cohort. Culture-positive patients in their study had higher leukocyte and procalcitonin concentrations, greater APACHE II and PIRO scores, longer hospitalization, and higher in-hospital mortality than culture-negative patients [20]. The higher RIN-IM scores among culture-positive patients in our study are consistent with this observation because the score integrates inflammatory cell indices with renal, metabolic, and nutritional components. However, our adjusted association persisted after controlling for nonrenal organ dysfunction and several factors affecting culture yield, suggesting that the observed relationship was not attributable solely to greater conventional illness severity. Therefore, the observed association should be interpreted as an association with the probability of microbiological confirmation under local sampling and laboratory practices rather than as evidence of a specific culture-positive biological phenotype.

Afzal et al. included 23,973 patients in a meta-analysis comparing culture-positive and culture-negative sepsis. They found no significant differences in mortality, mechanical ventilation, renal replacement therapy, or ICU length of stay, although culture-positive patients had a longer overall hospital stay [21]. These results emphasize that microbiological confirmation does not invariably identify a population with worse outcomes and should not be regarded as an independent measure of sepsis severity. Accordingly, the outcome gradients observed in our study were evaluated across RIN-IM categories and should not be interpreted as evidence that culture positivity itself caused increased mortality.

Meena et al. prospectively evaluated 633 patients with sepsis and reported blood-culture positivity in 41.9%. Although culture-positive patients presented with greater illness severity, blood-culture positivity was not independently associated with 30-day mortality [22]. Their findings support the distinction between microbiological detection and prognosis. In our cohort, the RIN-IM score may therefore represent a broader host-response and physiological-severity construct rather than a direct indicator of viable microbial burden. A patient can have a high score because of severe inflammation and organ dysfunction despite negative cultures, whereas a patient with a localized or early infection may have positive cultures without a markedly elevated score.

The observed association should not be interpreted as evidence that RIN-IM is biologically linked to microbial burden, pathogen viability, or antimicrobial-resistance mechanisms. RIN-IM integrates inflammatory cell indices with renal, nutritional, and metabolic variables, all of which may reflect the overall physiological disturbance accompanying critical illness. Accordingly, its association with microbiological confirmation may arise because systemic inflammation, organ dysfunction, infection source, healthcare exposure, antimicrobial treatment, and sampling practices coexist within the same clinical episode. The score cannot distinguish whether a higher value reflects infection-related host response, chronic comorbidity, treatment effects, or noninfectious critical illness and therefore cannot be used as a surrogate measure of pathogen burden.

Jiang et al. analyzed 16,007 critically ill patients with sepsis and demonstrated a nonlinear association between admission SII and mortality, supporting the ability of neutrophil-, lymphocyte-, and platelet-derived indices to reflect clinically relevant immune dysregulation [23]. Liang et al. subsequently pooled nine cohort studies comprising 25,626 patients and found that elevated SII was associated with a 51% higher risk of short-term mortality [10]. Although these studies primarily investigated prognosis rather than culture positivity, their findings support the inclusion of SII as a marker of the intensity and imbalance of the systemic immune response. In our study, the addition of metabolic, renal, and nutritional variables may have extended this prognostic information toward microbiological and resistance-related phenotypes.

Cao et al. evaluated 5357 ICU patients with sepsis and found a nonlinear inverse association between serum albumin and short- and long-term mortality; each 1-g/dL increase in albumin was associated with a substantial reduction in adjusted mortality risk, particularly at albumin concentrations below 2.6 g/dL [24]. Hypoalbuminemia in sepsis may reflect systemic inflammation, impaired hepatic synthesis, endothelial leakage, renal loss, malnutrition, and dilution following fluid administration. Its contribution to the RIN-IM score may therefore integrate both acute pathophysiology and underlying physiological reserve. Nevertheless, albumin is not infection-specific and may also be reduced by chronic liver disease, malignancy, nephrotic protein loss, and pre-existing malnutrition.

Zheng et al. studied 1257 critically ill patients with sepsis and demonstrated that a higher TyG index was independently associated with increased in-hospital, 28-day, and ICU mortality [25]. This association was interpreted as evidence that insulin resistance and disturbed glucose–lipid metabolism are linked to sepsis severity. The TyG component of the RIN-IM score may similarly capture the metabolic consequences of catecholamine release, inflammatory signaling, insulin resistance, exogenous glucose administration, and altered lipid utilization. However, TyG values can be influenced by diabetes, nutritional support, fasting status, corticosteroid treatment, and glucose-lowering therapy. Moreover, fasting status, same-phlebotomy sampling, and the timing of glucose and triglyceride measurements relative to resuscitation or early metabolic interventions could not be uniformly verified. Therefore, the TyG component should be interpreted as an admission-based marker of acute metabolic status rather than a validated measure of chronic insulin resistance.

Bayraktar et al. examined PIV in 96 older ICU patients with sepsis and found that PIV alone did not discriminate 28-day mortality, with an AUC of 0.550 [17]. This contrasting result is important because it illustrates the context dependence of individual hematologic indices, particularly in older patients affected by immunosenescence, chronic inflammation, bone marrow dysfunction, or immunosuppressive treatment. Our findings do not imply that PIV alone can identify culture positivity or resistant organisms. Instead, they support the hypothesis that combining PIV with SII, creatinine, albumin, and TyG may compensate partially for the limitations of individual biomarkers.

The RIN-IM score achieved an AUC of 0.752 for culture-positive sepsis. This represents moderate rather than excellent discrimination and indicates that the score can enrich the probability of culture positivity but cannot definitively confirm or exclude it. Gómez et al. reported an AUC of 0.67 for procalcitonin when microbiological confirmation from all evaluable culture types was considered in patients with sepsis [26]. Although the RIN-IM score showed numerically greater discrimination in our cohort, direct comparisons should be made cautiously because of differences in populations, culture definitions, specimen types, infection sources, antimicrobial exposure, and analytical methods. A formal head-to-head comparison with procalcitonin, C-reactive protein, lactate, and established severity scores would be needed to determine whether RIN-IM provides incremental diagnostic value.

The fixed RIN-IM score showed an apparent AUC of 0.752 for culture-positive sepsis in the present cohort. Because the score components and point thresholds had been specified in the previous development study and were not recalibrated or optimized here, this estimate should be interpreted as an apparent validation-cohort discrimination measure rather than a bootstrap-corrected estimate of model-development optimism. Bootstrap correction was instead applied to the newly fitted clinical reference and clinical-plus-RIN-IM models used in the incremental-performance analysis. In those resamples, all regression coefficients were refitted, whereas the RIN-IM score and its predefined thresholds remained fixed. These internal analyses do not establish transportability; external validation is required before the score or its categories can be considered for use in other settings.

Pre-culture antimicrobial exposure is an important potential source of differential microbiological yield. In routine care, microbiological specimens should be obtained before antimicrobial treatment whenever feasible; however, empirical treatment should not be delayed or discontinued in patients with suspected sepsis or septic shock. In this retrospective cohort, antimicrobial exposure before culture collection was defined as receipt of at least one systemic antibacterial dose within the preceding 48 h and was included as a prespecified covariate in the primary adjusted model. In a sensitivity analysis restricted to patients without pre-culture antimicrobial exposure (n = 141; 84 culture-positive cases), the association between the RIN-IM score and culture-positive sepsis remained directionally consistent (adjusted OR per one-point increase, 1.42; 95% CI, 1.23–1.65; high- versus low-risk category, adjusted OR, 5.36; 95% CI, 2.08–13.81). Nevertheless, the timing, spectrum, and duration of previous antimicrobial treatment may not have been fully captured in retrospective records and residual confounding cannot be excluded.

A secondary exploratory finding was that, among patients with culture-positive sepsis, higher RIN-IM scores were associated with MDR and XDR phenotypes. Because these analyses were conditioned on microbiological confirmation, they describe associations within a selected culture-positive subgroup and do not estimate the probability of resistant infection among all patients with sepsis. The MDR association was observed for the continuous score and for the high-risk category, whereas the XDR analysis was based on only 20 events and yielded imprecise categorical estimates. The four PDR infections were insufficient for reliable inferential analysis and were described only descriptively. Maia et al. found an MDRO prevalence of 9.8% among 864 critically ill patients admitted with sepsis and demonstrated that MDRO infection was associated with longer ICU and hospital stays and increased adjusted hospital mortality [27]. Their reported prevalence was lower than the MDR proportion in our culture-positive subgroup. However, the denominator and clinical setting differed substantially: Maia et al. evaluated resistant infection at hospital admission among all patients with sepsis, whereas our MDR proportion was calculated among patients with microbiologically confirmed infection and included both community- and hospital-onset episodes. Local antimicrobial exposure and resistance ecology may also explain part of this difference.

Gram-negative organisms predominated in our cohort, with *Klebsiella pneumoniae*, *Acinetobacter baumannii*, *Escherichia coli*, and *Pseudomonas aeruginosa* accounting for a substantial proportion of the isolates. Approximately one-third of Gram-negative isolates were carbapenem-resistant. Kumar et al. reported that 62% of nosocomial bloodstream infections in an ICU cohort were caused by Gram-negative organisms and identified carbapenem-resistant *A. baumannii* as the most prevalent pathogen [28]. The concordance between these pathogen distributions supports the clinical relevance of the resistance pattern observed in our cohort, while also emphasizing that organism prevalence is highly dependent on local ICU ecology.

Naghavi et al. estimated that bacterial AMR was associated with approximately 4.71 million deaths and directly attributable to approximately 1.14 million deaths worldwide in 2021. They also identified *S. aureus*, *A. baumannii*, *E. coli*, *K. pneumoniae*, *S. pneumoniae*, and *P. aeruginosa* as the leading contributors to the global AMR burden and reported a marked increase in mortality attributable to carbapenem-resistant Gram-negative organisms [7]. Four of these globally important pathogens were also among the predominant organisms in our cohort. This overlap underscores the clinical importance of antimicrobial resistance in critically ill patients; however, it does not establish that a host-response score can identify resistance mechanisms or determine the likelihood of a specific resistance phenotype while susceptibility results are pending.

The association between RIN-IM and MDR/XDR infection should not be interpreted as evidence that the score directly detects resistance mechanisms. Resistant infection is influenced by previous antimicrobial treatment, healthcare exposure, invasive procedures, colonization history, duration of hospitalization, infection source, and local clonal transmission. A high RIN-IM score may identify patients with greater inflammatory and physiological derangement who are also more likely to have healthcare-associated or high-burden infections. Although our adjusted analyses accounted for several important covariates, residual confounding by prior hospitalization, duration and spectrum of antimicrobial exposure, invasive devices, and previous colonization cannot be excluded. Accordingly, RIN-IM should complement—not replace—individual risk-factor assessment and the local antibiogram.

The moderate AUCs for MDR and XDR infection also require cautious interpretation. The AUC of 0.724 for MDR infection indicates moderate within-cohort discrimination but remains insufficient for selecting empirical antimicrobials as a standalone measure. The AUC of 0.742 for XDR infection was based on only 20 events and had a comparatively wide confidence interval. It should therefore be regarded as exploratory until validated in substantially larger populations. In particular, the low absolute number of XDR and PDR infections increases the risk of unstable estimates and exaggerated apparent performance.

The graded association between RIN-IM categories and clinical outcomes strengthens the clinical coherence of the score. Twenty-eight-day mortality increased from 13.6% in the low-risk category to 27.7% in the intermediate-risk category and 47.9% in the high-risk category. The corresponding proportions requiring mechanical ventilation were 36.4%, 56.9%, and 74.6%, while vasopressor requirements increased from 29.1% to 53.3% and 77.5%. ICU and hospital lengths of stay also increased progressively. Kayan et al. previously demonstrated a pronounced and monotonic 28-day mortality gradient across the same RIN-IM categories in the original development cohort [9]. The present study reproduced the direction of this mortality gradient in a geographically independent, sepsis-specific ICU cohort, although mortality was a secondary outcome and the current analysis was not designed as a formal external validation of the original mortality model. Jiang et al., Liang et al., Cao et al., and Zheng et al. independently demonstrated prognostic relationships for SII, albumin, and TyG in sepsis populations [10,23,24,25]. The present results extend these observations by showing that an integrated score derived from these biological domains is associated not only with adverse outcomes but also with microbiological confirmation and resistance phenotypes. Nevertheless, because the outcome comparisons across risk categories were primarily descriptive, dedicated adjusted survival analyses would be required before claiming that RIN-IM independently predicts mortality.

From a clinical perspective, the present retrospective associations do not establish clinical utility for RIN-IM. The score should not be used to determine whether cultures are obtained, to infer a specific pathogen or resistance phenotype, or to select empirical antimicrobial therapy. Appropriate blood cultures and site-specific microbiological specimens should be obtained in all patients with suspected sepsis, ideally before antimicrobial therapy when this does not delay treatment, irrespective of the RIN-IM score [4]. Prospective multicenter studies should evaluate whether RIN-IM adds information beyond established clinical risk factors and local microbiological data and whether any score-informed strategy improves diagnostic processes or patient outcomes.

Rapszky et al. analyzed 75 studies involving 11,393 patients and found that rapid molecular assays had a pooled patient-level sensitivity of 65.9% and specificity of 85.8% compared with blood culture [29]. They concluded that molecular assays may complement but cannot currently replace conventional cultures. The same principle applies to the present findings: RIN-IM cannot provide organism identification, distinguish infection from colonization in isolation, or determine antimicrobial susceptibility. Its observed association with culture positivity does not establish patient-level pretest-probability estimation or diagnostic-prioritization utility. Definitive microbiological testing remains essential for antimicrobial de-escalation and targeted treatment.

This study has several strengths. It evaluated a readily obtainable score based on routinely measured laboratory variables in a real-world ICU population. Culture-positive sepsis, MDR infection, and XDR infection were analyzed separately, and resistance analyses distinguished patient-level outcomes from isolate-level microbiological descriptions. The primary culture-positivity model adjusted for factors likely to affect microbiological yield and severity, including pre-culture antimicrobial exposure, hospital-onset infection, immunosuppression, infection source, and nonrenal SOFA score. The use of nonrenal SOFA reduced, although did not eliminate, potential mathematical overlap between creatinine in the RIN-IM score and renal dysfunction within SOFA. Sensitivity analyses using alternative adjustment strategies yielded consistent findings, and bootstrap-based evaluation addressed internal optimism and calibration.

Several limitations should also be acknowledged. First, the retrospective, single-center design is susceptible to selection bias, measurement error, residual confounding, and limits generalizability. Second, culture-negative sepsis is not equivalent to absence of microbial infection. Previous antimicrobial therapy, inadequate sampling, low circulating microbial burden, fastidious organisms, viral infection, noncultivable pathogens, and infections confined to poorly sampled anatomical sites may have produced false-negative cultures. Although pre-culture antimicrobial exposure was included in adjusted analyses and examined in a restriction-based sensitivity analysis, incomplete ascertainment of its timing, spectrum, and duration may have contributed to residual confounding. Third, when pre-infection organ-function data were unavailable, the baseline SOFA score was assumed to be zero unless chronic organ dysfunction was documented. Incomplete documentation of chronic renal, respiratory, cardiovascular, hepatic, neurological, or coagulation dysfunction may therefore have caused some chronic abnormalities to be misclassified as acute organ dysfunction. This could have led to erroneous fulfilment of the required acute SOFA increase and, consequently, misclassification of Sepsis-3 eligibility at cohort entry. Such misclassification may have influenced the composition of the analytic cohort and the observed associations. Sensitivity analysis using total SOFA addressed model adjustment but could not resolve uncertainty regarding baseline organ function or possible misclassification of Sepsis-3 eligibility. Fungal and bacterial infections differ in epidemiology, host response, diagnostic yield, and clinical course, which may have added biological heterogeneity to the composite outcome. However, exclusion of fungal culture-positive cases produced a materially unchanged association, supporting the robustness of the primary finding. Fourth, SII and PIV are mathematically related because both are derived from overlapping neutrophil, platelet, and lymphocyte components, while PIV additionally incorporates monocytes. In the exploratory component-level analysis, the two indices showed a strong positive correlation (Spearman’s ρ = 0.840, *p* < 0.001), with VIF values of 3.72 for ln(SII) and 3.68 for ln(PIV) (Supplementary Table S5). Although these VIF values did not exceed the prespecified threshold for potentially important multicollinearity, the correlation indicates measurable biological and mathematical redundancy within the inflammatory domain. Importantly, the primary multivariable analyses entered the total RIN-IM score alone, rather than SII and PIV as separate covariates. Therefore, the reported association should be interpreted as that of a composite host-response score and not as evidence of independent effects of its individual inflammatory components. Fifth, the score components are influenced by conditions unrelated to acute infection. Creatinine varies with chronic kidney disease, age, muscle mass, and fluid balance; albumin is influenced by chronic liver disease, nutrition, renal protein loss, and fluid administration; and TyG varies with diabetes, feeding status, corticosteroids, insulin treatment, and lipid-lowering therapy. Sixth, single-time-point laboratory values cannot capture the dynamic evolution of immune, renal, and metabolic dysfunction. Serial RIN-IM measurements may provide additional information but were not examined. In addition, the timing of RIN-IM assessment and microbiological sampling was not fully synchronized. The score was calculated from the earliest eligible laboratory values obtained within 24 h after ICU admission, whereas specimens related to the index sepsis episode could be collected from 48 h before to 72 h after the documented index time. Therefore, particularly in hospital-onset infections, the admission RIN-IM score may have preceded the inflammatory and metabolic state at the time of culture collection by a clinically meaningful interval. This temporal separation may have attenuated or distorted the observed association and precludes interpretation of RIN-IM as a real-time predictor of microbiological positivity. Future prospective studies should measure the score at a prespecified time relative to suspected sepsis and obtain microbiological specimens contemporaneously. Seventh, antimicrobial-resistance epidemiology, microbiological sampling practices, and empirical antimicrobial prescribing patterns are center specific. Although cumulative antibiograms are important stewardship tools, they do not reliably identify resistance at the individual-patient level. Hasegawa et al. analyzed 1,484,038 *Escherichia coli* and 671,035 *Klebsiella* spp. isolates from 127 Veterans Health Administration facilities and found that hospital-level antibiograms had limited discrimination for patient-level resistance, with area under the curve values ranging from 0.576 to 0.715 across antimicrobial agents [30]. These findings indicate that local antibiograms should inform but cannot replace assessment of individual patient factors such as prior antimicrobial exposure, healthcare contact, previous colonization, infection source, and severity of illness. Reports of sustained high carbapenem-resistant *Pseudomonas aeruginosa* prevalence in specific regional settings further illustrate that local resistance ecology may differ substantially across healthcare systems. Accordingly, the performance, calibration, and clinical utility of the RIN-IM score for MDR or XDR risk enrichment may not transport unchanged to settings with different pathogen distributions, resistance prevalence, laboratory practices, or antibiograms. Multicenter external validation across diverse resistance settings is required before any score threshold is used to inform empirical antimicrobial decisions [30,31]. Eighth, the MDR and XDR analyses were restricted to the 154 patients with culture-positive sepsis. Because culture positivity was associated with RIN-IM and was also influenced by sampling practices, prior antimicrobial exposure, infection source, healthcare exposure, and illness severity, conditioning the analyses on microbiological confirmation may have introduced selection/collider bias. Accordingly, the MDR and XDR estimates describe exploratory associations within a microbiologically selected subgroup and should not be interpreted as estimates of resistant-infection risk or as evidence that RIN-IM predicts resistant infection in the overall sepsis population. This restriction also limits direct comparison of resistance-related risk between culture-positive and culture-negative patients. In addition, the XDR analysis was based on only 20 events and produced wide confidence intervals despite use of Firth’s bias-reduced logistic regression; its estimates should therefore be considered exploratory. The very small number of PDR infections prevented reliable modeling. Ninth, some patients contributed more than one isolate. Therefore, all isolate-level organism and resistance findings are presented descriptively and should not be interpreted as independent comparative estimates; patient-level analyses are more appropriate for clinical inference. Although the present cohort was geographically independent of the original RIN-IM development cohort, this study was primarily designed to investigate culture positivity and antimicrobial-resistance phenotypes rather than to perform a complete external validation of the original mortality model. Specifically, recalibration of the original mortality equation, assessment of calibration-in-the-large, and decision-curve analysis for mortality were not undertaken. Therefore, additional temporal and geographical validation remain necessary before the score or its predefined categories can be used to guide diagnostic, prognostic, or antimicrobial decisions. In addition, classification of microbiological results as clinically significant infection, contamination, or colonization required clinical judgment. Although two investigators independently reviewed each result using prespecified criteria and resolved discrepancies by consensus, they were not blinded to routinely available laboratory values in the clinical record. The calculated RIN-IM score and risk category were unavailable during adjudication; nevertheless, partial awareness of its component values and the absence of a formal inter-rater agreement estimate may have contributed to outcome misclassification. Finally, 131 of the 449 patients who fulfilled the Sepsis-3 criteria were excluded before construction of the analytic cohort. Although the exclusion criteria were prespecified and are detailed in the flow diagram, 49 patients had no valid microbiological specimen related to the index episode, 38 had one or more missing RIN-IM components, 27 represented repeat ICU admissions, and 17 had incomplete essential records. These exclusions may have preferentially retained patients who underwent more complete early laboratory evaluation and microbiological investigation and may therefore have selected a subgroup with different illness severity, care intensity, sampling practices, antimicrobial exposure, or outcome risk than the full Sepsis-3 population. The direction and magnitude of any resulting selection bias cannot be determined from the available data. Accordingly, the reported associations apply to this selected cohort with calculable early RIN-IM measurements and clinically interpretable microbiological assessment and may not generalize to all ICU patients meeting Sepsis-3 criteria.

## 5. Conclusions

In this single-center retrospective cohort, higher admission RIN-IM scores were associated with culture-positive sepsis. Among patients with culture-positive sepsis, higher RIN-IM scores were associated with MDR infection; however, this analysis was conditioned on microbiological confirmation and does not estimate resistant-infection risk in the overall sepsis population. The association with XDR infection was exploratory, was based on only 20 events, and requires confirmation in substantially larger independent cohorts. These findings do not demonstrate that RIN-IM predicts microbiological positivity, identifies pathogens or resistance mechanisms, quantifies microbial burden, establishes a direct biological relationship with resistance, or provides clinical utility for antimicrobial selection.

The observed associations may also have been influenced by uncertainty in baseline organ function and possible misclassification of acute SOFA change and Sepsis-3 eligibility when previous organ-function data were unavailable. The observed associations may have been influenced by residual confounding, temporal separation between laboratory assessment and microbiological sampling, heterogeneity of specimen types, local sampling and laboratory practices, and restriction of resistance analyses to culture-positive patients. RIN-IM should not be used to determine whether microbiological cultures are obtained; to guide microbiological decision-making; or to select, broaden, or withhold empirical antimicrobial therapy. Prospective multicenter studies with time-aligned measurements, comparison with established clinical risk factors and local microbiological data, external validation, and clinical-impact assessment are required before any clinical implementation is considered.

## Figures and Tables

**Table 1:** Complete calculation algorithm and derivation of the simplified RIN-IM Score [9].

Domain	Component	0 Points	1 Point	2 Points	Possible Domain Score
Renal	Serum creatinine (mg/dL)	<1.50	1.50–1.99	≥2.00	0–2
Inflammatory	SII	<1000	1000–2999	≥3000	0–4 *
Inflammatory	PIV	<500	500–1199	≥1200	0–4 *
Nutritional/protein status	Serum albumin (g/dL)	≥3.50	2.50–3.49	<2.50	0–2
Metabolic	TyG index	<9.00	9.00–9.49	≥9.50	0–2
Total RIN-IM Score					0–10

* The inflammatory domain includes both SII and PIV; therefore, its combined possible range is 0–4 points. SII = neutrophil count × platelet count/lymphocyte count; PIV = neutrophil count × monocyte count × platelet count/lymphocyte count; TyG index = ln[(triglycerides [mg/dL] × glucose [mg/dL])/2]. Complete blood-count components are expressed in ×10^9^/L. The total RIN-IM Score was calculated by summing the five component scores. Low-, intermediate-, and high-risk categories are defined as 0–3, 4–6, and 7–10 points, respectively.

**Table 2:** Baseline demographic and clinical characteristics of the study population according to culture status.

Characteristic	Overall (N = 318)	Culture-Negative (n = 164)	Culture-Positive (n = 154)	*p*-Value
**Demographic characteristics**				
Age, years	69.2 ± 12.3	68.4 ± 12.8	70.1 ± 11.7	0.217 ᵃ
Male sex	182 (57.2)	91 (55.5)	91 (59.1)	0.516 ᵇ
**Infection-related characteristics**				
Hospital-onset infection	133 (41.8)	58 (35.4)	75 (48.7)	0.016 ᵇ
Antimicrobial exposure before culture collection	177 (55.7)	107 (65.2)	70 (45.5)	<0.001 ᵇ
Suspected infection source				0.046 ᵇ
Respiratory tract	129 (40.6)	78 (47.6)	51 (33.1)	
Urinary tract	64 (20.1)	30 (18.3)	34 (22.1)	
Intra-abdominal	46 (14.5)	19 (11.6)	27 (17.5)	
Bloodstream/catheter-related	26 (8.2)	8 (4.9)	18 (11.7)	
Skin and soft tissue	22 (6.9)	12 (7.3)	10 (6.5)	
Other or undetermined	31 (9.7)	17 (10.4)	14 (9.1)	
Central venous catheter present	197 (61.9)	92 (56.1)	105 (68.2)	0.027 ᵇ
**Comorbid conditions**				
Diabetes mellitus	130 (40.9)	64 (39.0)	66 (42.9)	0.487 ᵇ
Hypertension	188 (59.1)	98 (59.8)	90 (58.4)	0.812 ᵇ
Chronic kidney disease	70 (22.0)	31 (18.9)	39 (25.3)	0.167 ᵇ
Chronic cardiovascular disease	134 (42.1)	66 (40.2)	68 (44.2)	0.480 ᵇ
Chronic respiratory disease	76 (23.9)	43 (26.2)	33 (21.4)	0.317 ᵇ
Malignancy	55 (17.3)	25 (15.2)	30 (19.5)	0.318 ᵇ
Immunosuppression	47 (14.8)	18 (11.0)	29 (18.8)	0.049 ᵇ
**Illness severity at index assessment**				
Septic shock	119 (37.4)	52 (31.7)	67 (43.5)	0.030 ᵇ
SOFA score	8 [5–11]	7 [5–10]	9 [6–12]	<0.001 ᶜ
Non-renal SOFA score	7 [4–9]	6 [4–8]	7 [5–10]	0.002 ᶜ
APACHE II score ᵈ	19 [14–25]	18 [13–23]	21 [15–27]	0.004 ᶜ
Serum lactate, mmol/L	2.7 [1.6–4.7]	2.4 [1.5–4.1]	3.1 [1.8–5.2]	0.015 ᶜ
Invasive mechanical ventilation at index assessment	151 (47.5)	71 (43.3)	80 (51.9)	0.122 ᵇ

Data are presented as mean ± standard deviation, median [interquartile range], or n (%). Percentages were calculated within the corresponding culture-status column. Culture positivity was defined by the recovery of at least one clinically significant microorganism within the predefined sepsis-related sampling window. ᵃ Independent-samples *t*-test. ᵇ Pearson’s chi-square test. ᶜ Mann–Whitney *U* test. ᵈ APACHE II scores were available for 291 patients (149 culture-negative and 142 culture-positive). APACHE II, Acute Physiology and Chronic Health Evaluation II; SOFA, Sequential Organ Failure Assessment.

**Table 3:** Laboratory components and distribution of the RIN-IM score according to culture status.

Variable	Overall (N = 318)	Culture-Negative (n = 164)	Culture-Positive (n = 154)	*p*-Value
**Hematological variables**				
White blood cell count, ×10^9^/L	13.8 [9.4–18.9]	12.6 [8.8–17.2]	15.1 [10.3–20.8]	0.001 ᵃ
Neutrophil count, ×10^9^/L	11.6 [7.6–16.3]	10.4 [6.9–14.7]	13.0 [8.7–18.4]	<0.001 ᵃ
Lymphocyte count, ×10^9^/L	0.94 [0.62–1.39]	1.08 [0.73–1.51]	0.80 [0.52–1.20]	<0.001 ᵃ
Monocyte count, ×10^9^/L	0.66 [0.42–0.93]	0.61 [0.39–0.87]	0.71 [0.46–1.01]	0.018 ᵃ
Platelet count, ×10^9^/L	224 [163–302]	218 [162–289]	232 [166–315]	0.276 ᵃ
**Biochemical variables**				
Creatinine, mg/dL	1.43 [0.98–2.10]	1.26 [0.90–1.82]	1.64 [1.09–2.46]	0.002 ᵃ
Albumin, g/dL	2.93 [2.48–3.37]	3.12 [2.72–3.55]	2.72 [2.30–3.17]	<0.001 ᵃ
Glucose, mg/dL	157 [119–220]	145 [113–198]	171 [128–239]	0.006 ᵃ
Triglycerides, mg/dL	151 [108–207]	139 [101–189]	165 [117–226]	0.004 ᵃ
**Derived RIN-IM indices**				
SII	2860 [1480–5250]	2110 [1090–3910]	3825 [2210–6910]	<0.001 ᵃ
PIV	1810 [820–3690]	1280 [590–2540]	2680 [1370–5290]	<0.001 ᵃ
TyG index	9.37 [8.98–9.76]	9.19 [8.84–9.57]	9.55 [9.15–9.91]	<0.001 ᵃ
**RIN-IM component scores**				
Creatinine component, points	0 [0–2]	0 [0–1]	1 [0–2]	0.002 ᵃ
SII component, points	1 [1–2]	1 [1–2]	2 [1–2]	<0.001 ᵃ
PIV component, points	2 [1–2]	2 [1–2]	2 [2–2]	<0.001 ᵃ
Albumin component, points	1 [1–2]	1 [0–1]	1 [1–2]	<0.001 ᵃ
TyG component, points	1 [0–2]	1 [0–1]	1 [1–2]	<0.001 ᵃ
**Total RIN-IM score**	5 [3–7]	4 [2–5]	6 [4–8]	<0.001 ᵃ
**RIN-IM risk category**				<0.001 ᵇ
Low risk (0–3 points)	110 (34.6)	78 (47.6)	32 (20.8)	
Intermediate risk (4–6 points)	137 (43.1)	68 (41.5)	69 (44.8)	
High risk (7–10 points)	71 (22.3)	18 (11.0)	53 (34.4)	

Data are presented as median [interquartile range] or n (%). Percentages in the culture-status columns were calculated using the corresponding column total. Culture-positive sepsis was present in 29.1% of patients in the low-risk group, 50.4% in the intermediate-risk group, and 74.6% in the high-risk group (*p* for trend < 0.001). PIV, pan-immune inflammation value; RIN-IM, renal–inflammatory–nutritional–metabolic; SII, systemic immune-inflammation index; TyG, triglyceride–glucose index. ᵃ Mann–Whitney *U* test. ᵇ Pearson’s chi-square test for the overall distribution of RIN-IM risk categories. TyG was calculated as ln[(triglycerides [mg/dL] × glucose [mg/dL])/2] using the earliest available values within 24 h of ICU admission; fasting status was neither required nor assumed.

**Table 4:** Patient-level microbiological characteristics and descriptive isolate-level antimicrobial resistance profiles among patients with culture-positive sepsis.

Microbiological Characteristic	Overall N = 154	Low Risk (n = 32)	Intermediate risk (n = 69)	High Risk (n = 53)	*p*-Value
**Patient-level characteristics, n (%)**					
Primary positive specimen type					0.858 ᵃ
Blood	54 (35.1)	8 (25.0)	23 (33.3)	23 (43.4)	
Respiratory tract specimen	45 (29.2)	10 (31.3)	20 (29.0)	15 (28.3)	
Urine	31 (20.1)	8 (25.0)	14 (20.3)	9 (17.0)	
Intra-abdominal specimen	12 (7.8)	2 (6.3)	6 (8.7)	4 (7.5)	
Wound or soft-tissue specimen	7 (4.5)	2 (6.3)	4 (5.8)	1 (1.9)	
Other normally sterile specimen	5 (3.2)	2 (6.3)	2 (2.9)	1 (1.9)	
Polymicrobial infection	17 (11.0)	2 (6.3)	8 (11.6)	7 (13.2)	0.686 ᵇ
**Isolate-level characteristics, n (%)**	171 isolates	34 isolates	77 isolates	60 isolates	
Microorganism group					—
Gram-negative bacteria	122 (71.3)	23 (67.6)	53 (68.8)	46 (76.7)	
Gram-positive bacteria	42 (24.6)	9 (26.5)	21 (27.3)	12 (20.0)	
Fungi	7 (4.1)	2 (5.9)	3 (3.9)	2 (3.3)	
**Gram-negative bacteria**					
*Klebsiella pneumoniae*	36 (21.1)	5 (14.7)	16 (20.8)	15 (25.0)	—
*Acinetobacter baumannii*	29 (17.0)	3 (8.8)	11 (14.3)	15 (25.0)	—
*Escherichia coli*	25 (14.6)	7 (20.6)	11 (14.3)	7 (11.7)	—
*Pseudomonas aeruginosa*	17 (9.9)	3 (8.8)	8 (10.4)	6 (10.0)	—
*Enterobacter cloacae* complex	7 (4.1)	2 (5.9)	3 (3.9)	2 (3.3)	—
Other Gram-negative bacteria	8 (4.7)	3 (8.8)	4 (5.2)	1 (1.7)	—
**Gram-positive bacteria**					
*Staphylococcus aureus*	13 (7.6)	3 (8.8)	6 (7.8)	4 (6.7)	—
Coagulase-negative staphylococci	10 (5.8)	3 (8.8)	5 (6.5)	2 (3.3)	—
*Enterococcus faecium*	9 (5.3)	1 (2.9)	5 (6.5)	3 (5.0)	—
*Enterococcus faecalis*	5 (2.9)	1 (2.9)	2 (2.6)	2 (3.3)	—
*Streptococcus* species	3 (1.8)	1 (2.9)	2 (2.6)	0 (0.0)	—
Other Gram-positive bacteria	2 (1.2)	0 (0.0)	1 (1.3)	1 (1.7)	—
**Fungi**					
*Candida albicans*	4 (2.3)	1 (2.9)	2 (2.6)	1 (1.7)	—
Non-*albicans Candida* species	3 (1.8)	1 (2.9)	1 (1.3)	1 (1.7)	—
**Resistance classifications among bacterial isolates**	n = 164	n = 32	n = 74	n = 58	
MDR, including XDR and PDR	72 (43.9)	8 (25.0)	30 (40.5)	34 (58.6)	—
XDR, including PDR	21 (12.8)	1 (3.1)	8 (10.8)	12 (20.7)	—
PDR	4 (2.4)	0 (0.0)	1 (1.4)	3 (5.2)	—
**Selected resistance phenotypes**					
Carbapenem-resistant Gram-negative bacteria ᶜ	38/122 (31.1)	4/23 (17.4)	15/53 (28.3)	19/46 (41.3)	—
ESBL-producing Enterobacterales ᵈ	31/68 (45.6)	4/14 (28.6)	13/30 (43.3)	14/24 (58.3)	—
Methicillin-resistant *S. aureus* ᵉ	6/13 (46.2)	1/3 (33.3)	2/6 (33.3)	3/4 (75.0)	—
Vancomycin-resistant *Enterococcus* spp. ᶠ	4/14 (28.6)	0/2 (0.0)	2/7 (28.6)	2/5 (40.0)	—

ESBL, extended-spectrum β-lactamase; MDR, multidrug-resistant; PDR, pandrug-resistant; XDR, extensively drug-resistant. Isolate-level organism and resistance data are presented descriptively only. Because some patients contributed multiple isolates, no inferential comparisons were performed for isolate-level variables. ᵃ Fisher–Freeman–Halton exact test. ᵇ Exact test for sparse categorical data. ᶜ Denominator: Gram-negative bacterial isolates. ᵈ Denominator: *E. coli*, *K. pneumoniae*, and *E. cloacae* complex isolates. ᵉ Denominator: *S. aureus* isolates. ᶠ Denominator: *E. faecium* and *E. faecalis* isolates. Patient-level analyses used 318 patients overall or 154 patients with culture-positive sepsis, as specified. Isolate-level descriptive analyses used 171 nonduplicate clinically significant isolates. Antimicrobial-susceptibility and isolate-level resistance descriptions were restricted to 164 bacterial isolates. Patient-level MDR and XDR outcomes were defined among the 154 culture-positive patients; a patient was classified according to the most resistant phenotype identified during the index sepsis episode. Percentages were calculated using the denominator specified for each section or row.

**Table 5:** Incremental performance of prespecified models for culture-positive sepsis.

Model	Apparent AUC (95% CI)	Bootstrap- Corrected AUC	Brier Score	Calibration Intercept	Calibration Slope	ΔAUC vs. Reference Model	*p*-Value
Clinical reference model using non-renal SOFA	0.748 (0.695–0.801)	0.742	0.201	−0.04	0.92	Reference	—
Clinical reference model plus RIN-IM score	0.799 (0.751–0.847)	0.791	0.181	−0.02	0.94	0.051	0.006
Clinical reference model using total SOFA	0.753 (0.701–0.805)	0.747	0.198	−0.05	0.91	Reference	—
Clinical reference model plus RIN-IM score	0.794 (0.746–0.842)	0.787	0.184	−0.03	0.93	0.041	0.018

Table *p*-values are from DeLong tests comparing correlated AUCs. Likelihood-ratio *p*-values for addition of RIN-IM were <0.001 for the non-renal SOFA model and 0.002 for the total-SOFA sensitivity model. Bootstrap-corrected performance was estimated using 1000 bootstrap resamples for the newly fitted clinical reference and clinical-plus-RIN-IM models. In each resample, the intercept and all regression coefficients were refitted. The RIN-IM score itself, its component thresholds, and its predefined risk-category cutoffs were held fixed and were not recalculated or optimized. Likelihood-ratio *p*-values for addition of RIN-IM were <0.001 for the non-renal SOFA model and 0.002 for the total-SOFA sensitivity model. Clinical reference models included age, sex, antimicrobial exposure before culture collection, hospital-onset infection, immunosuppression, and infection source, together with either non-renal SOFA (primary analysis) or total SOFA (sensitivity analysis). RIN-IM was entered as a continuous score per one-point increase. AUC, area under the receiver operating characteristic curve; CI, confidence interval; RIN-IM, renal-inflammatory-nutritional-metabolic score; SOFA, Sequential Organ Failure Assessment.

**Table 6:** Discriminatory performance of the RIN-IM score for culture-positive sepsis and antimicrobial resistance phenotypes.

Performance Measure	Culture-Positive Sepsis	MDR Infection ᶜ	XDR Infection ᵈ
Analysis population, n	318	154	154
Events, n (%)	154 (48.4)	67 (43.5)	20 (13.0)
Apparent AUC (95% CI)	0.752 (0.699–0.805)	0.724 (0.643–0.805)	0.742 (0.635–0.849)
Bootstrap-corrected AUC ᵃ	0.748	0.716	0.729
*p*-value for AUC > 0.50	<0.001	<0.001	<0.001
Exploratory cut-off ᵇ	≥5	≥6	≥7
Sensitivity, % (95% CI)	68.8 (61.1–75.6)	70.1 (58.3–79.8)	60.0 (38.7–78.1)
Specificity, % (95% CI)	70.1 (62.7–76.6)	67.8 (57.4–76.7)	69.4 (61.2–76.6)
PPV, % (95% CI)	68.4 (60.7–75.2)	62.7 (51.4–72.7)	22.6 (13.5–35.5)
NPV, % (95% CI)	70.6 (63.1–77.0)	74.7 (64.1–83.0)	92.1 (85.1–95.9)
Youden index	0.390	0.380	0.294

ᵃ Optimism-corrected AUCs were estimated using 1000 bootstrap resamples. ᵇ Cut-off values were selected by maximizing the Youden index and should be considered exploratory rather than clinically validated decision thresholds. ᶜ MDR analyses were performed at the patient level among culture-positive patients. Patients with at least one MDR, XDR, or PDR isolate were classified as having an MDR infection. ᵈ Patients with a PDR isolate were included in the XDR outcome. PDR was not evaluated separately because only four events occurred. AUC, area under the receiver operating characteristic curve; CI, confidence interval; MDR, multidrug-resistant; NPV, negative predictive value; PDR, pandrug-resistant; PPV, positive predictive value; RIN-IM, renal–inflammatory–nutritional–metabolic; XDR, extensively drug-resistant. Confidence intervals for sensitivity, specificity, PPV, and NPV were calculated using the Wilson method.

**Table 7:** Clinical outcomes according to the predefined RIN-IM risk categories.

Clinical Outcome	Overall (N = 318)	Low Risk (n = 110)	Intermediate Risk (n = 137)	High Risk (n = 71)	*p*-Value
**Mortality outcomes**					
28-day mortality	87 (27.4)	15 (13.6)	38 (27.7)	34 (47.9)	<0.001 ᵃ
ICU mortality	72 (22.6)	12 (10.9)	31 (22.6)	29 (40.8)	<0.001 ᵃ
In-hospital mortality	81 (25.5)	14 (12.7)	35 (25.5)	32 (45.1)	<0.001 ᵃ
**Organ-support requirements**					
Invasive mechanical ventilation during ICU stays	171 (53.8)	40 (36.4)	78 (56.9)	53 (74.6)	<0.001 ᵃ
Vasopressor requirement during ICU stays	160 (50.3)	32 (29.1)	73 (53.3)	55 (77.5)	<0.001 ᵃ
**Duration and resource-use outcomes**					
Duration of invasive mechanical ventilation, days ᵇ	7 [3–13]	4 [2–7]	7 [4–12]	10 [6–17]	<0.001 ᶜ
ICU length of stay, days	9 [5–15]	6 [4–9]	9 [6–14]	13 [8–21]	<0.001 ᶜ
Hospital length of stay, days	16 [10–25]	12 [8–18]	17 [11–25]	22 [14–33]	<0.001 ᶜ

ICU, intensive care unit; RIN-IM, renal–inflammatory–nutritional–metabolic. Data are presented as n (%) or median [interquartile range]. Percentages were calculated using the corresponding RIN-IM category total.; ᵃ Cochran–Armitage test for trend across increasing RIN-IM risk categories.; ᵇ Duration of invasive mechanical ventilation was calculated only among mechanically ventilated patients: overall, n = 171; low risk, n = 40; intermediate risk, n = 78; and high risk, n = 53.; ᶜ Kruskal–Wallis test. The organ-support variables indicate the requirement at any time during the ICU stay and should not be confused with support being administered at the index assessment.

## Data Availability

The original contributions presented in this study are included in the article.

## Supplementary Materials

The following supporting information can be downloaded at: https://www.mdpi.com/article/10.3390/diagnostics16203350/s1, Table S1: Completeness and missingness of variables included in the analyses. Table S2: Detailed organism-specific antimicrobial susceptibility and resistance profiles. Table S3: Complete univariable logistic regression analyses for culture-positive sepsis and MDR infection. Table S4: Sensitivity analyses of the association between the RIN-IM score and culture-positive sepsis. Table S5. Pairwise correlation and collinearity diagnostics for the inflammatory components of the RIN-IM score. Figure S1: Calibration plot of the multivariable model for culture-positive sepsis. Figure S2: Bootstrap-corrected discrimination and calibration performance of the primary model.

## Abbreviations

AMR	Antimicrobial resistance
aOR	Adjusted odds ratio
APACHE II	Acute Physiology and Chronic Health Evaluation II
AST	Antimicrobial susceptibility testing
IQR	Interquartile range
CI	Confidence interval
CLSI	Clinical and Laboratory Standards Institute
CRP	C-reactive protein
ESBL	Extended-spectrum β-lactamase
EUCAST	European Committee on Antimicrobial Susceptibility Testing
ICU	Intensive care unit
PIV	Pan-immune inflammation value
PPV	Positive predictive value
RIN-IM	Renal–inflammatory–nutritional–metabolic score
ROC	Receiver operating characteristic
SD	Standard deviation
SII	Systemic immune-inflammation index
SOFA	Sequential Organ Failure Assessment
MDR	Multidrug-resistant
NPV	Negative predictive value
OR	Odds ratio
PDR	Pandrug-resistant
AUC	Area under the receiver operating characteristic curve
STROBE	Strengthening the Reporting of Observational Studies in Epidemiology
