# Supplementary material for: From Host Response to Microbiological Risk: Association of the RIN-IM Score with Culture-Positive Sepsis and Antimicrobial Resistance in Critically Ill Adults
Source: Diagnostics (Basel). 2026 Oct 14;16(20):3350. doi: 10.3390/diagnostics16203350 (PMC13702883; doi:10.3390/diagnostics16203350)
Supplement: Supplementary file 1 [file diagnostics-16-03350-s001.zip › diagnostics-4517287-supplementary.pdf]

Supplementary Table S1. Completeness and missingness of variables included in the analyses.

Variable or data domain	Relevant population	Available, n (%)	Missing, n (%)	Handling in the analyses
**Demographic and clinical variables**				
Age	318 patients	318 (100.0)	0 (0.0)	Included
Sex	318 patients	318 (100.0)	0 (0.0)	Included
Comorbid conditions	318 patients	318 (100.0)	0 (0.0)	Included
Immunosuppression status	318 patients	318 (100.0)	0 (0.0)	Included
Hospital- or community-onset infection	318 patients	318 (100.0)	0 (0.0)	Included
Suspected infection source	318 patients	318 (100.0)	0 (0.0)	Included
Pre-culture antimicrobial exposure	318 patients	318 (100.0)	0 (0.0)	Included
Central venous catheter status	318 patients	318 (100.0)	0 (0.0)	Included
Septic shock status	318 patients	318 (100.0)	0 (0.0)	Included
**Illness-severity variables**				
SOFA score	318 patients	318 (100.0)	0 (0.0)	Included in descriptive analyses
Non-renal SOFA score	318 patients	318 (100.0)	0 (0.0)	Included in primary adjusted models
APACHE II score	318 patients	291 (91.5)	27 (8.5)	Available-case sensitivity analysis
Serum lactate	318 patients	318 (100.0)	0 (0.0)	Included
**RIN-IM component variables**				
Creatinine	318 patients	318 (100.0)	0 (0.0)	Required for RIN-IM calculation
Albumin	318 patients	318 (100.0)	0 (0.0)	Required for RIN-IM calculation
Neutrophil count	318 patients	318 (100.0)	0 (0.0)	Required for SII and PIV
Lymphocyte count	318 patients	318 (100.0)	0 (0.0)	Required for SII and PIV
Monocyte count	318 patients	318 (100.0)	0 (0.0)	Required for PIV
Platelet count	318 patients	318 (100.0)	0 (0.0)	Required for SII and PIV
Glucose	318 patients	318 (100.0)	0 (0.0)	Required for TyG calculation
Triglycerides	318 patients	318 (100.0)	0 (0.0)	Required for TyG calculation
SII	318 patients	318 (100.0)	0 (0.0)	Derived variable
PIV	318 patients	318 (100.0)	0 (0.0)	Derived variable
TyG index	318 patients	318 (100.0)	0 (0.0)	Derived variable
Total RIN-IM score	318 patients	318 (100.0)	0 (0.0)	Primary exposure variable
RIN-IM risk category	318 patients	318 (100.0)	0 (0.0)	Primary categorical exposure
**Microbiological variables**				
Culture status	318 patients	318 (100.0)	0 (0.0)	Primary outcome
Primary positive specimen type	154 culture-positive patients	154 (100.0)	0 (0.0)	Included
Microorganism identification	171 clinically significant isolates	171 (100.0)	0 (0.0)	Included
Antimicrobial susceptibility results	164 bacterial isolates	164 (100.0)	0 (0.0)	Included
MDR classification	164 bacterial isolates	164 (100.0)	0 (0.0)	Included
XDR classification	164 bacterial isolates	164 (100.0)	0 (0.0)	Included

Variable or data domain	Relevant population	Available, n (%)	Missing, n (%)	Handling in the analyses
PDR classification	164 bacterial isolates	164 (100.0)	0 (0.0)	Descriptive analysis only
Patient-level MDR status	154 culture-positive patients	154 (100.0)	0 (0.0)	Secondary regression analysis
Patient-level XDR status	154 culture-positive patients	154 (100.0)	0 (0.0)	Firth logistic regression
**Clinical outcomes**				
28-day mortality	318 patients	318 (100.0)	0 (0.0)	Included
ICU mortality	318 patients	318 (100.0)	0 (0.0)	Included
In-hospital mortality	318 patients	318 (100.0)	0 (0.0)	Included
Invasive mechanical ventilation	318 patients	318 (100.0)	0 (0.0)	Included
Vasopressor requirement	318 patients	318 (100.0)	0 (0.0)	Included
Duration of mechanical ventilation[a]	171 ventilated patients	171 (100.0)	0 (0.0)	Included
ICU length of stay	318 patients	318 (100.0)	0 (0.0)	Included
Hospital length of stay	318 patients	318 (100.0)	0 (0.0)	Included

No statistical imputation was performed. Primary analyses were based on complete observations for the variables included in each model. The APACHE II sensitivity analysis was restricted to the 291 patients with an available APACHE II score. [a] Patients who did not receive invasive mechanical ventilation were considered structurally inapplicable rather than missing. APACHE II, Acute Physiology and Chronic Health Evaluation II; ICU, intensive care unit; MDR, multidrug-resistant; PDR, pandrug-resistant; PIV, pan-immune inflammation value; RIN-IM, renal–inflammatory–nutritional–immunometabolic; SII, systemic immune-inflammation index; SOFA, Sequential Organ Failure Assessment; TyG, triglyceride–glucose index; XDR, extensively drug-resistant.

Supplementary Table S2. Detailed organism-specific antimicrobial susceptibility and resistance profiles.

Organism and antimicrobial agent	Isolates tested, n	S, n (%)	I, n (%)	R, n (%)
Klebsiella pneumoniae **(n = 36)**				
└ Piperacillin–tazobactam	36	15 (41.7)	3 (8.3)	18 (50.0)
└ Ceftriaxone	36	14 (38.9)	1 (2.8)	21 (58.3)
└ Cefepime	36	17 (47.2)	2 (5.6)	17 (47.2)
└ Meropenem	36	22 (61.1)	1 (2.8)	13 (36.1)
└ Amikacin	36	25 (69.4)	2 (5.6)	9 (25.0)
└ Gentamicin	36	22 (61.1)	1 (2.8)	13 (36.1)
└ Ciprofloxacin	36	16 (44.4)	2 (5.6)	18 (50.0)
└ Trimethoprim–sulfamethoxazole	36	18 (50.0)	0 (0.0)	18 (50.0)
└ Colistin	18	16 (88.9)	0 (0.0)	2 (11.1)
Escherichia coli **(n = 25)**				
└ Piperacillin–tazobactam	25	17 (68.0)	2 (8.0)	6 (24.0)
└ Ceftriaxone	25	15 (60.0)	0 (0.0)	10 (40.0)
└ Cefepime	25	16 (64.0)	1 (4.0)	8 (32.0)
└ Meropenem	25	23 (92.0)	0 (0.0)	2 (8.0)
└ Amikacin	25	22 (88.0)	1 (4.0)	2 (8.0)
└ Gentamicin	25	17 (68.0)	1 (4.0)	7 (28.0)
└ Ciprofloxacin	25	11 (44.0)	2 (8.0)	12 (48.0)
└ Trimethoprim–sulfamethoxazole	25	13 (52.0)	0 (0.0)	12 (48.0)
└ Colistin	5	5 (100.0)	0 (0.0)	0 (0.0)
Enterobacter cloacae **complex (n = 7)**				
└ Piperacillin–tazobactam	7	3 (42.9)	1 (14.3)	3 (42.9)

Organism and antimicrobial agent	Isolates tested, n	S, n (%)	I, n (%)	R, n (%)
└ Ceftriaxone	7	2 (28.6)	0 (0.0)	5 (71.4)
└ Cefepime	7	4 (57.1)	0 (0.0)	3 (42.9)
└ Meropenem	7	6 (85.7)	0 (0.0)	1 (14.3)
└ Amikacin	7	6 (85.7)	0 (0.0)	1 (14.3)
└ Gentamicin	7	5 (71.4)	0 (0.0)	2 (28.6)
└ Ciprofloxacin	7	4 (57.1)	1 (14.3)	2 (28.6)
└ Trimethoprim–sulfamethoxazole	7	4 (57.1)	0 (0.0)	3 (42.9)
***Acinetobacter baumannii* (n = 29)**				
└ Ampicillin–sulbactam	29	8 (27.6)	2 (6.9)	19 (65.5)
└ Cefepime	29	6 (20.7)	2 (6.9)	21 (72.4)
└ Meropenem	29	9 (31.0)	1 (3.4)	19 (65.5)
└ Amikacin	29	12 (41.4)	2 (6.9)	15 (51.7)
└ Gentamicin	29	10 (34.5)	1 (3.4)	18 (62.1)
└ Ciprofloxacin	29	5 (17.2)	1 (3.4)	23 (79.3)
└ Trimethoprim–sulfamethoxazole	29	9 (31.0)	0 (0.0)	20 (69.0)
└ Colistin	24	21 (87.5)	0 (0.0)	3 (12.5)
***Pseudomonas aeruginosa* (n = 17)**				
└ Piperacillin–tazobactam	17	10 (58.8)	1 (5.9)	6 (35.3)
└ Ceftazidime	17	11 (64.7)	1 (5.9)	5 (29.4)
└ Cefepime	17	11 (64.7)	1 (5.9)	5 (29.4)
└ Meropenem	17	13 (76.5)	1 (5.9)	3 (17.6)
└ Amikacin	17	15 (88.2)	0 (0.0)	2 (11.8)
└ Ciprofloxacin	17	10 (58.8)	1 (5.9)	6 (35.3)
└ Colistin	8	8 (100.0)	0 (0.0)	0 (0.0)
***Staphylococcus aureus* (n = 13)**				
└ Cefoxitin	13	7 (53.8)	0 (0.0)	6 (46.2)
└ Clindamycin	13	8 (61.5)	0 (0.0)	5 (38.5)
└ Erythromycin	13	6 (46.2)	1 (7.7)	6 (46.2)
└ Levofloxacin	13	7 (53.8)	1 (7.7)	5 (38.5)
└ Trimethoprim–sulfamethoxazole	13	10 (76.9)	0 (0.0)	3 (23.1)
└ Vancomycin	13	13 (100.0)	0 (0.0)	0 (0.0)
└ Linezolid	13	13 (100.0)	0 (0.0)	0 (0.0)
**Coagulase-negative staphylococci (n = 10)**				
└ Cefoxitin	10	4 (40.0)	0 (0.0)	6 (60.0)
└ Clindamycin	10	5 (50.0)	0 (0.0)	5 (50.0)
└ Erythromycin	10	4 (40.0)	0 (0.0)	6 (60.0)
└ Levofloxacin	10	6 (60.0)	0 (0.0)	4 (40.0)
└ Vancomycin	10	10 (100.0)	0 (0.0)	0 (0.0)
└ Linezolid	10	10 (100.0)	0 (0.0)	0 (0.0)
***Enterococcus faecium* (n = 9)**				
└ Ampicillin	9	2 (22.2)	0 (0.0)	7 (77.8)
└ Vancomycin	9	6 (66.7)	0 (0.0)	3 (33.3)
└ Teicoplanin	9	6 (66.7)	0 (0.0)	3 (33.3)
└ Linezolid	9	8 (88.9)	0 (0.0)	1 (11.1)
***Enterococcus faecalis* (n = 5)**				
└ Ampicillin	5	5 (100.0)	0 (0.0)	0 (0.0)
└ Vancomycin	5	4 (80.0)	0 (0.0)	1 (20.0)

Organism and antimicrobial agent	Isolates tested, n	S, n (%)	I, n (%)	R, n (%)
└ Teicoplanin	5	4 (80.0)	0 (0.0)	1 (20.0)
└ Linezolid	5	5 (100.0)	0 (0.0)	0 (0.0)
***Streptococcus* species (n = 3)**				
└ Penicillin	3	3 (100.0)	0 (0.0)	0 (0.0)
└ Ceftriaxone	3	3 (100.0)	0 (0.0)	0 (0.0)
└ Erythromycin	3	2 (66.7)	0 (0.0)	1 (33.3)
└ Vancomycin	3	3 (100.0)	0 (0.0)	0 (0.0)
***Candida albicans* (n = 4)**				
└ Fluconazole	4	4 (100.0)	0 (0.0)	0 (0.0)
└ Voriconazole	4	4 (100.0)	0 (0.0)	0 (0.0)
└ Micafungin	4	4 (100.0)	0 (0.0)	0 (0.0)
└ Amphotericin B	4	4 (100.0)	0 (0.0)	0 (0.0)
**Non-*albicans Candida* species (n = 3)**				
└ Fluconazole	3	1 (33.3)	1 (33.3)	1 (33.3)
└ Voriconazole	3	2 (66.7)	0 (0.0)	1 (33.3)
└ Micafungin	3	3 (100.0)	0 (0.0)	0 (0.0)
└ Amphotericin B	3	3 (100.0)	0 (0.0)	0 (0.0)

Susceptibility categories were interpreted according to EUCAST clinical breakpoint tables applicable at the time of testing.

Supplementary Table S3. Complete univariable logistic regression analyses for culture-positive sepsis and MDR infection.

Predictor	Culture-positive sepsis, unadjusted OR (95% CI)	p-value	MDR infection, unadjusted OR (95% CI)	p-value
**Demographic characteristics**				
Age, per 1-year increase	1.011 (0.994–1.029)	0.217	1.016 (0.987–1.045)	0.278
Male sex	1.16 (0.74–1.81)	0.516	1.30 (0.68–2.50)	0.426
**Infection-related characteristics**				
Hospital-onset infection	1.74 (1.11–2.72)	**0.016**	2.75 (1.42–5.31)	**0.003**
Antimicrobial exposure before culture collection	0.44 (0.28–0.70)	**<0.001**	2.25 (1.17–4.32)	**0.015**
Suspected infection source		**0.046**[a]		0.381[a]
└ Respiratory tract	Reference	—	Reference	—
└ Urinary tract	1.73 (0.95–3.17)	0.075	1.13 (0.46–2.79)	0.783
└ Intra-abdominal	2.17 (1.10–4.31)	**0.026**	1.47 (0.57–3.80)	0.430
└ Bloodstream/catheter-related	3.44 (1.39–8.50)	**0.007**	2.88 (0.95–8.73)	0.061
└ Skin and soft tissue	1.27 (0.51–3.17)	0.602	1.83 (0.47–7.19)	0.385
└ Other or undetermined	1.26 (0.57–2.78)	0.567	2.44 (0.73–8.15)	0.146
Central venous catheter present	1.68 (1.06–2.65)	**0.027**	1.95 (0.96–3.96)	0.065
**Comorbid conditions**				
Diabetes mellitus	1.17 (0.75–1.83)	0.487	1.43 (0.75–2.71)	0.281
Hypertension	0.95 (0.61–1.48)	0.812	1.37 (0.71–2.62)	0.349
Chronic kidney disease	1.46 (0.85–2.48)	0.168	2.01 (0.96–4.20)	0.062
Chronic cardiovascular disease	1.17 (0.75–1.83)	0.480	1.44 (0.76–2.74)	0.264
Chronic respiratory disease	0.77 (0.46–1.29)	0.317	1.11 (0.51–2.40)	0.799
Malignancy	1.35 (0.75–2.41)	0.319	1.64 (0.73–3.65)	0.229
Immunosuppression	1.88 (1.00–3.55)	0.051	2.13 (0.93–4.83)	0.072
**Illness-severity variables**				

Predictor	Culture-positive sepsis, unadjusted OR (95% CI)	p-value	MDR infection, unadjusted OR (95% CI)	p-value
Septic shock	1.66 (1.05–2.62)	**0.030**	2.62 (1.36–5.06)	**0.004**
SOFA score, per 1-point increase	1.21 (1.14–1.29)	**<0.001**	1.15 (1.07–1.24)	**<0.001**
Non-renal SOFA score, per 1-point increase	1.18 (1.11–1.26)	**<0.001**	1.13 (1.05–1.22)	**0.002**
APACHE II score, per 1-point increase[b]	1.06 (1.03–1.09)	**<0.001**	1.05 (1.01–1.09)	**0.009**
Serum lactate, per 1-mmol/L increase	1.10 (1.03–1.18)	**0.004**	1.09 (1.01–1.18)	**0.026**
**RIN-IM score**				
RIN-IM score, per 1-point increase	1.48 (1.35–1.63)	**<0.001**	1.38 (1.22–1.56)	**<0.001**

Each predictor was evaluated in a separate univariable logistic regression model. No covariate adjustment was applied in this table. The culture-positive sepsis analysis included all 318 patients, of whom 154 had culture-positive sepsis. The MDR analysis included 154 culture-positive patients, of whom 67 had at least one MDR, XDR, or PDR isolate. The categorical RIN-IM analyses are presented in Table 4 and were not repeated here.

[a] Global likelihood-ratio p-value for infection source. [b] APACHE II analyses were restricted to patients with available scores: 291 patients for culture-positive sepsis and 142 culture-positive patients for MDR infection.

APACHE II, Acute Physiology and Chronic Health Evaluation II; CI, confidence interval; MDR, multidrug-resistant; OR, odds ratio; PDR, pandrug-resistant; RIN-IM, renal–inflammatory–nutritional–immunometabolic; SOFA, Sequential Organ Failure Assessment; XDR, extensively drug-resistant.

Supplementary Table S4. Sensitivity analyses of the association between the RIN-IM score and culture-positive sepsis.

Analysis	Patients/ events, n/N	RIN-IM score, per 1-point increase, aOR (95% CI); p	Intermediate vs. low risk, aOR (95% CI); p	High vs. low risk, aOR (95% CI); p
Primary model using non-renal SOFA[a]	318/154	1.35 (1.21–1.51); **<0.001**	2.01 (1.13–3.57); **0.017**	4.65 (2.25–9.62); **<0.001**
Total SOFA substituted for non-renal SOFA[b]	318/154	1.31 (1.17–1.47); **<0.001**	1.90 (1.06–3.41); **0.031**	4.18 (2.00–8.73); **<0.001**
APACHE II substituted for non-renal SOFA[c]	291/142	1.34 (1.19–1.51); **<0.001**	1.96 (1.07–3.58); **0.029**	4.51 (2.11–9.65); **<0.001**
Patients without pre-culture antimicrobial exposure[d]	141/84	1.42 (1.23–1.65); **<0.001**	2.28 (1.05–4.95); **0.037**	5.36 (2.08–13.81); **<0.001**
Hospital-onset infection subgroup[e]	133/75	1.37 (1.17–1.61); **<0.001**	2.10 (0.91–4.83); 0.081	4.92 (1.73–13.99); **0.003**
Community-onset infection subgroup[e]	185/79	1.31 (1.14–1.51); **<0.001**	1.89 (0.91–3.94); 0.088	4.12 (1.62–10.49); **0.003**
Exclusion of fungal infections[f]	311/147	1.34 (1.20–1.50); **<0.001**	1.98 (1.11–3.53); **0.021**	4.52 (2.18–9.39); **<0.001**

The continuous and categorical representations of the RIN-IM score were evaluated in separate models. The low-risk category was used as the reference group in all categorical analyses.

[a] Adjusted for age, sex, antimicrobial exposure before culture collection, hospital-onset infection, immunosuppression, infection source, and non-renal SOFA score. [b] Adjusted for the same covariates as the primary model, with total SOFA replacing non-renal SOFA. [c] Restricted to the 291 patients with an available APACHE II score and adjusted for age, sex, pre-culture antimicrobial exposure, hospital-onset infection, immunosuppression, infection source, and APACHE II score. [d] Restricted to patients who had not received antimicrobial therapy before culture collection. The model was adjusted for age, sex, hospital-onset infection, immunosuppression, infection source, and non-renal SOFA score. [e] Stratified analyses were adjusted for age, sex, pre-culture antimicrobial exposure, immunosuppression, infection source, and non-renal SOFA score; hospital-onset status was omitted because it was constant within each subgroup. [f] Seven patients with fungal culture positivity were excluded. The adjustment set was identical to that of the primary model. All models converged

successfully, and no evidence of complete or quasi-complete separation was observed. The direction and magnitude of the associations remained broadly consistent across sensitivity analyses. aOR, adjusted odds ratio; APACHE II, Acute Physiology and Chronic Health Evaluation II; CI, confidence interval; RIN-IM, renal–inflammatory–nutritional–immunometabolic; SOFA, Sequential Organ Failure Assessment.

Supplementary Table S5. Pairwise correlation and collinearity diagnostics for the inflammatory components of the RIN-IM score.

**Analysis**	**Variables / model term**	**Statistic**	**Value**	**Interpretation**
Pairwise correlation	SII and PIV	Spearman's rho	0.840	Strong positive correlation
		p value	<0.001	
Exploratory component-level model*	ln(SII)	VIF	3.72	Moderate, but not potentially important collinearity
		Tolerance	0.269	
	ln(PIV)	VIF	3.68	Moderate, but not potentially important collinearity
		Tolerance	0.272	
	Remaining clinical covariates	VIF range	1.04–1.48	Low collinearity

*Binary logistic regression with culture-positive sepsis as the outcome. The model included ln(SII), ln(PIV), age, sex, antimicrobial exposure before culture collection, hospital-onset infection, immunosuppression, infection source, and non-renal SOFA score. The total RIN-IM score was not entered into this component-level model because it is mathematically derived from these biomarkers. In the prespecified primary analyses, only the total RIN-IM score—not SII and PIV as separate variables—was entered into the multivariable models. SII, systemic immune-inflammation index; PIV, pan-immune inflammation value; VIF, variance inflation factor. Pairwise correlations were assessed using Spearman's method. VIF >5 or tolerance <0.20 was prespecified as potentially important multicollinearity.

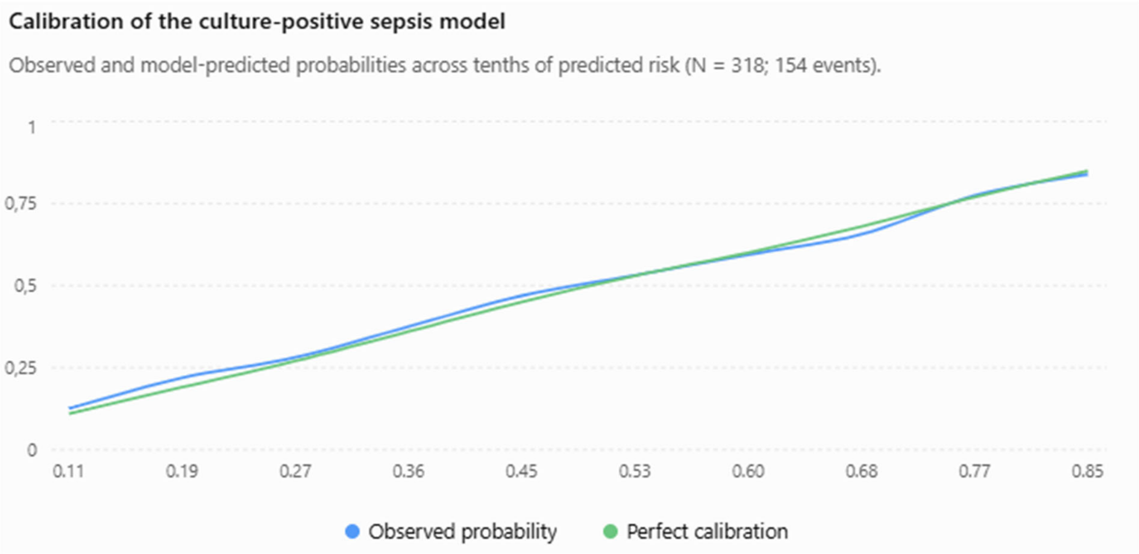

Supplementary Figure S1. Calibration of the multivariable model for culture-positive sepsis. The calibration plot compares model-predicted probabilities with the observed proportions of culture-positive sepsis among 318 patients, including 154 culture-positive events. Patients were grouped into tenths of predicted risk for graphical presentation. Circles represent the observed event proportion within each group, and the diagonal reference line indicates perfect agreement between predicted and observed probabilities. The calibration curve showed close agreement with the reference line across most of the predicted-risk range, with only minor deviations at intermediate and higher probabilities. The bootstrap-corrected calibration intercept was −0.04, the calibration slope was 0.92, and the Brier score was 0.194.

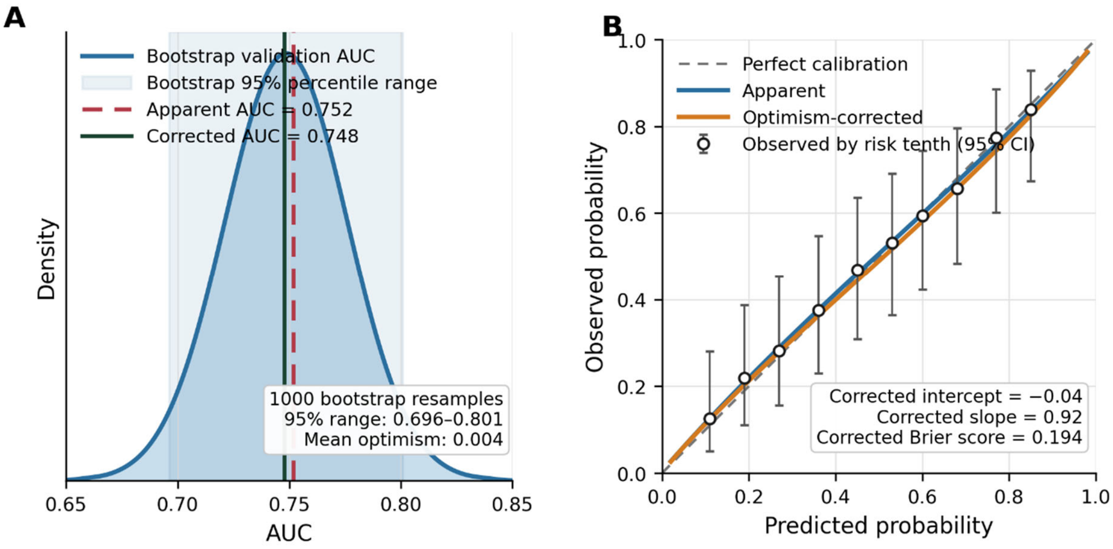

Supplementary Figure S2. Bootstrap-corrected discrimination and calibration performance of the primary model.
